# Supplementary material for: Changes in chromatin accessibility are not concordant with transcriptional changes for single‐factor perturbations
Source: Mol Syst Biol. 2022 Sep 7;18(9):e10979. doi: 10.15252/msb.202210979 (PMC9450098; doi:10.15252/msb.202210979)
Supplement: Supplementary file 1 — Appendix S1 [file MSB-18-e10979-s001.pdf]

Table of Contents

APPENDIX FIGURE S1 2

APPENDIX FIGURE S2 3

APPENDIX FIGURE S3 4

APPENDIX FIGURE S4 5

APPENDIX FIGURE S5 6

APPENDIX FIGURE S6 7

APPENDIX FIGURE S7 8

APPENDIX FIGURE S8 9

APPENDIX FIGURE S9 10

APPENDIX FIGURE S10 11

APPENDIX FIGURE S11 12

APPENDIX FIGURE S12 13

APPENDIX FIGURE S13 14

APPENDIX FIGURE S14 15

APPENDIX FIGURE S15 16

APPENDIX FIGURE S16 17

APPENDIX TABLE S1 18

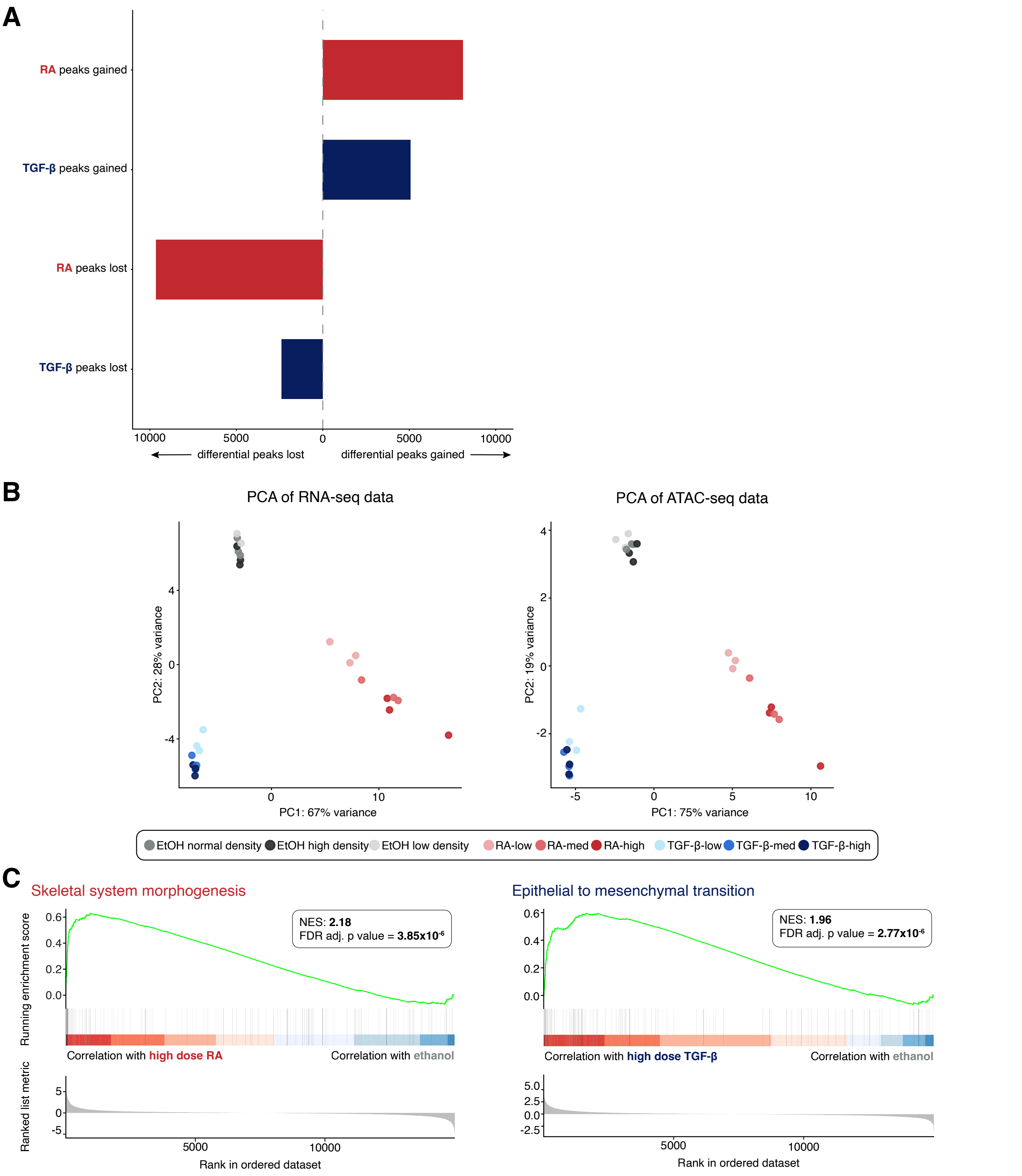

Appendix Figure S1. Global analysis of expression and chromatin accessibility changes in response to varying signals in MCF-7 cells. (A) The number of differentially accessible peaks for each dose of each condition compared to ethanol vehicle control further subsetted by those that increase in accessibility (right) and decrease in accessiblity (left) in response to signal. (B) PCA of variance stabilizing transformed raw counts from gene expression and chromatin accessibility data demonstrating the first two principal components. (C) Gene set enrichment analysis (GSEA) (Subramanian et al, 2005) of differentially expressed genes in response to high dose retinoic acid against a gene set for skeletal system morphogenesis. Genes whose expression were differentially expressed in response to TGF-β were enriched for genes associated with epithelial-to-mesenchymal transition. Green traces represent running enrichment scores across fold change ranked gene lists.

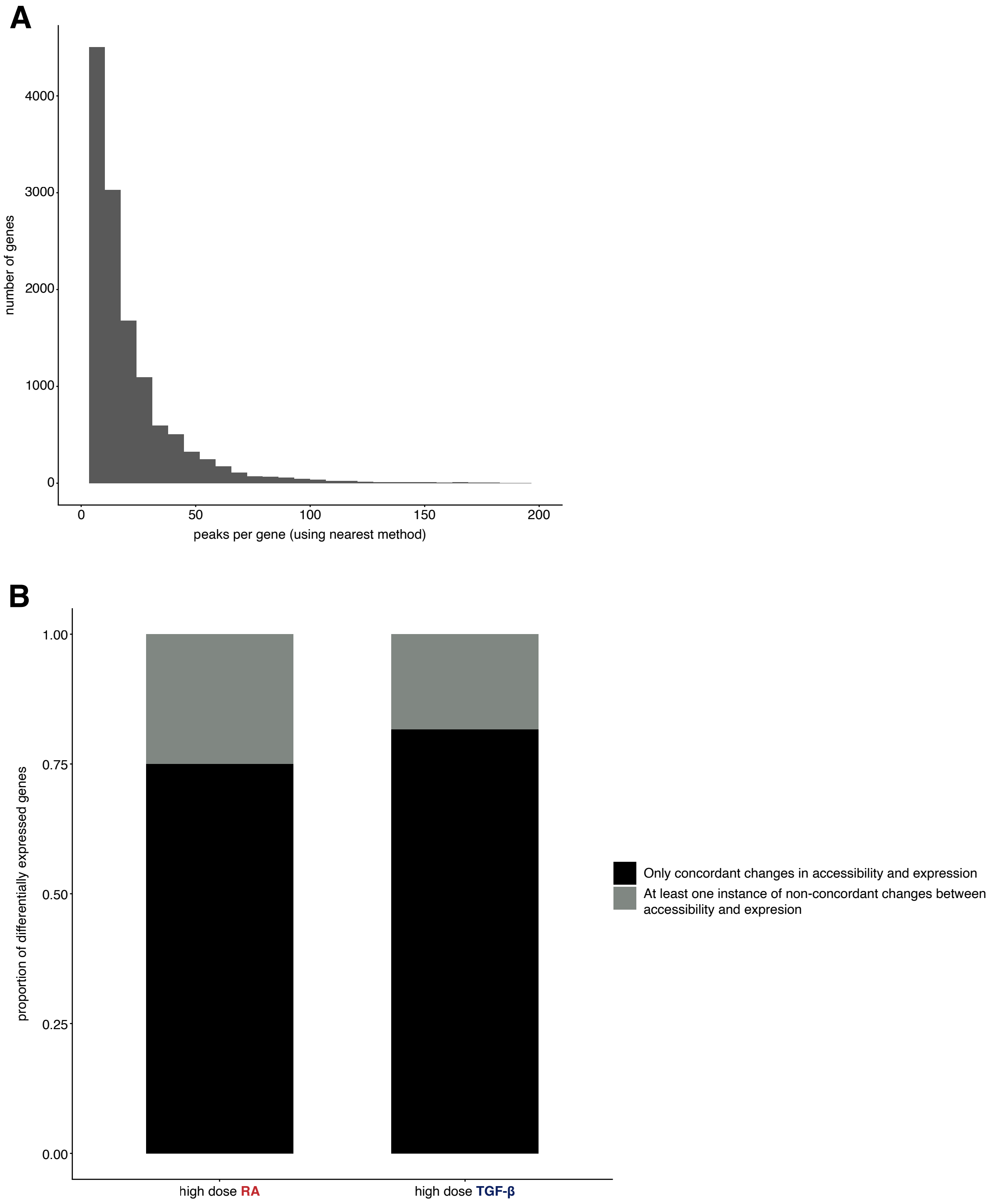

Appendix Figure S2. Further statistics of peaks/gene relationships using the 'nearest' method to assign peaks. (A) Histogram showing the distribution of peaks assigned per gene using the 'nearest' method of making peak/gene pairs. (B) Bar plot showing the proportion of differentially expressed genes in response to retinoic acid or TGF-β that had only differentially concordant peak accessiblity changes (i.e., increased accessiblity for increased expression or decreased accessibility for decreased expression, black) or at least one differentially accessible peak in the opposite direciton of the gene expression change (gray).

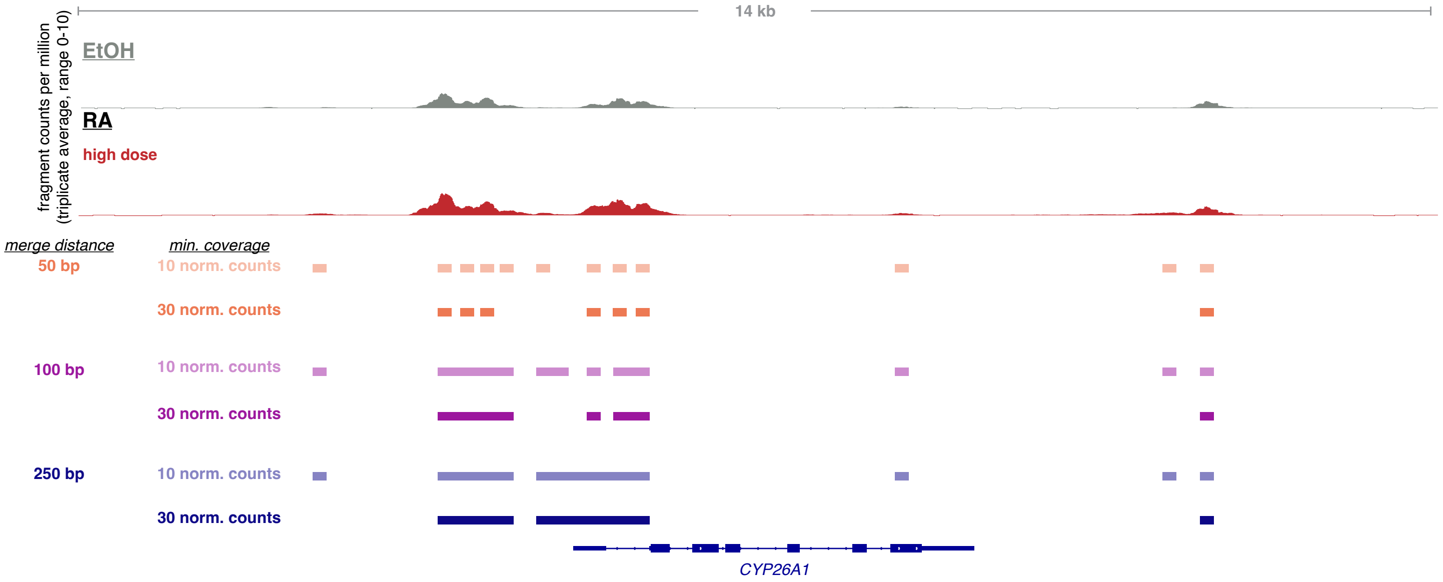

Appendix Figure S3. Tuning peak calling parameters. Representative peak calls at the CYP26A1 using different peak merge parameters (colors) and minimum normalized fragment count coverage (shades of the same color). Based on these results we selected a merge distance of 50 base pairs and a minimum coverage of 30 normalized fragment counts.

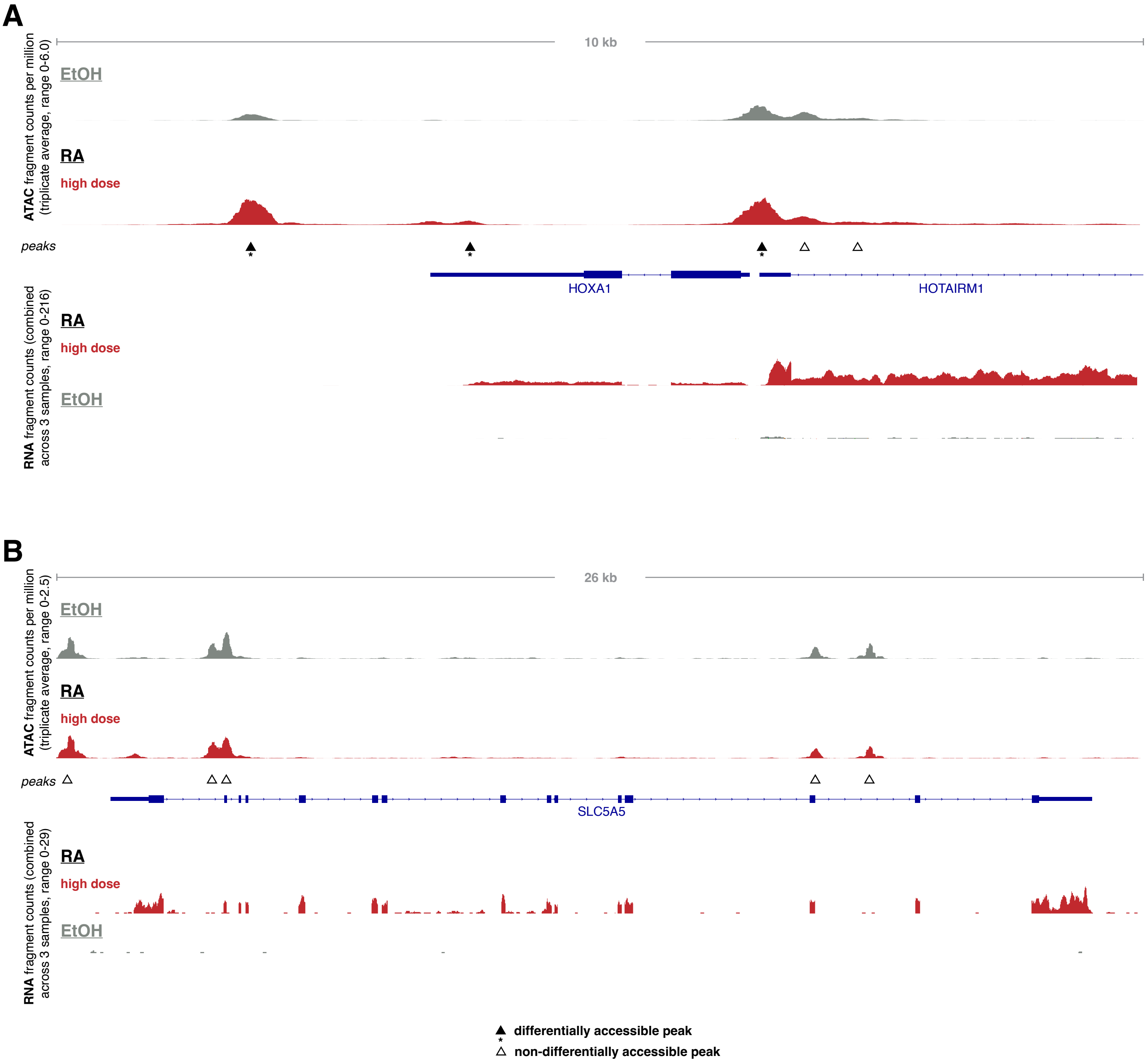

Appendix Figure S4. RNA fragment counts in response to high dose retinoic acid at *HOXA1* and *SLC5A5* loci. IGV tracks for *HOXA1* (A) and *SLC5A5* (B) showing the change in chromatin accessibility (top panels) and the distribution of final aligned RNA fragments (lower panels) before and after exposure to high dose retinoic acid. Peaks are denoted by arrowheads while peaks that are differentially accessible after exposure to high-dose retinoic acid are denoted with a black arrowhead with an asterisk beneath.

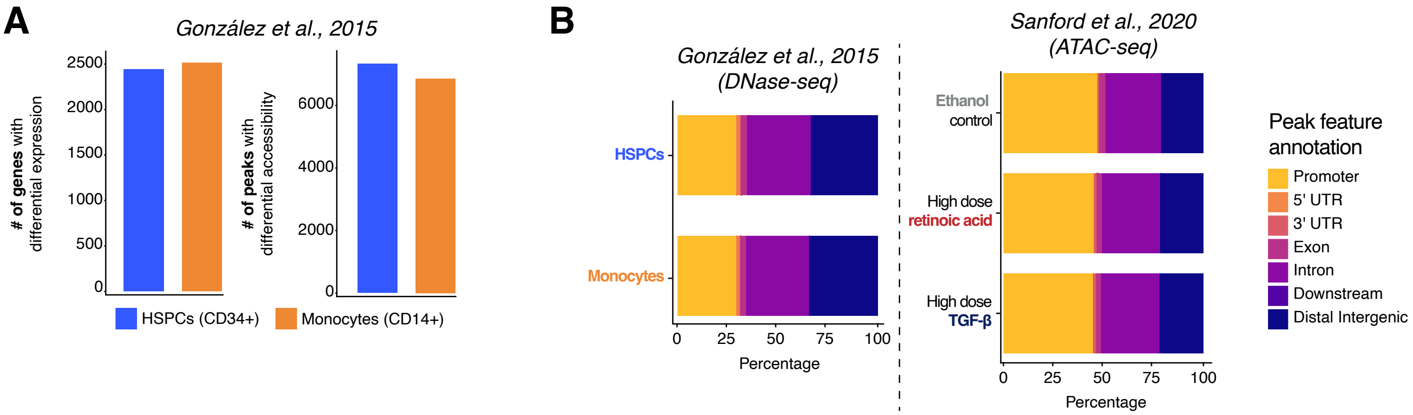

Appendix Figure S5. Comparison of accessibility data from hematopoietic differentiation and MCF-7 cells in response to signal. (A) Number of differentially expressed genes (left) specific to CD34+ hematopoietic stem and progenitor cells (HSPCs, blue) and CD14+ monocytes (orange) from data from González et al., 2015 and the number of differentially accessible peaks (DNase-seq) between the two populations (right). (B) Annotation of distribution of peak location in relation to gene transcriptional units for consensus files for HSPCs and monocytes (left). Distribution of accessible peak features for consensus peaks for MCF-7 cells in ethanol, high dose retinoic acid, and high dose TGF-β.

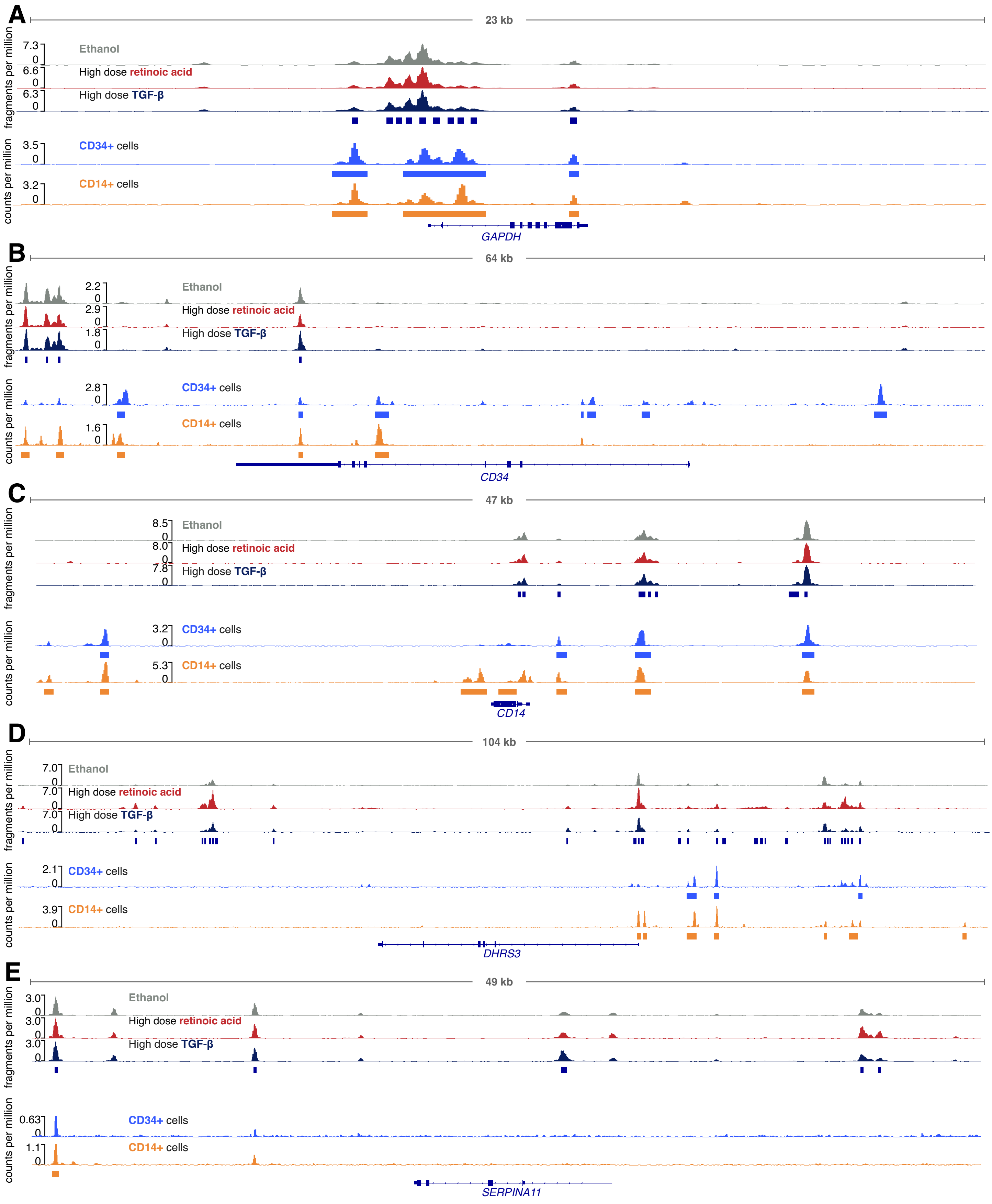

Appendix Figure S6. Comparison of peak calls at multiple loci across both hematopoietic differentiation and MCF-7 genome-wide accessibility data sets. Consensus peak calls for MCF-7 signal samples (ATAC-seq) and hematopoietic differentiation samples (DNase-seq) at a 'housekeeping' gene GAPDH (A), hematopoietic cell-specific marker loci CD34 (B) and CD14 (C), a retinoic acid responsive site, DHRS3 (D), and a TGF-β responsive site SERPINA11 (E). Values are fragments per million for ATAC-seq samples and counts per million for DNase-seq samples.

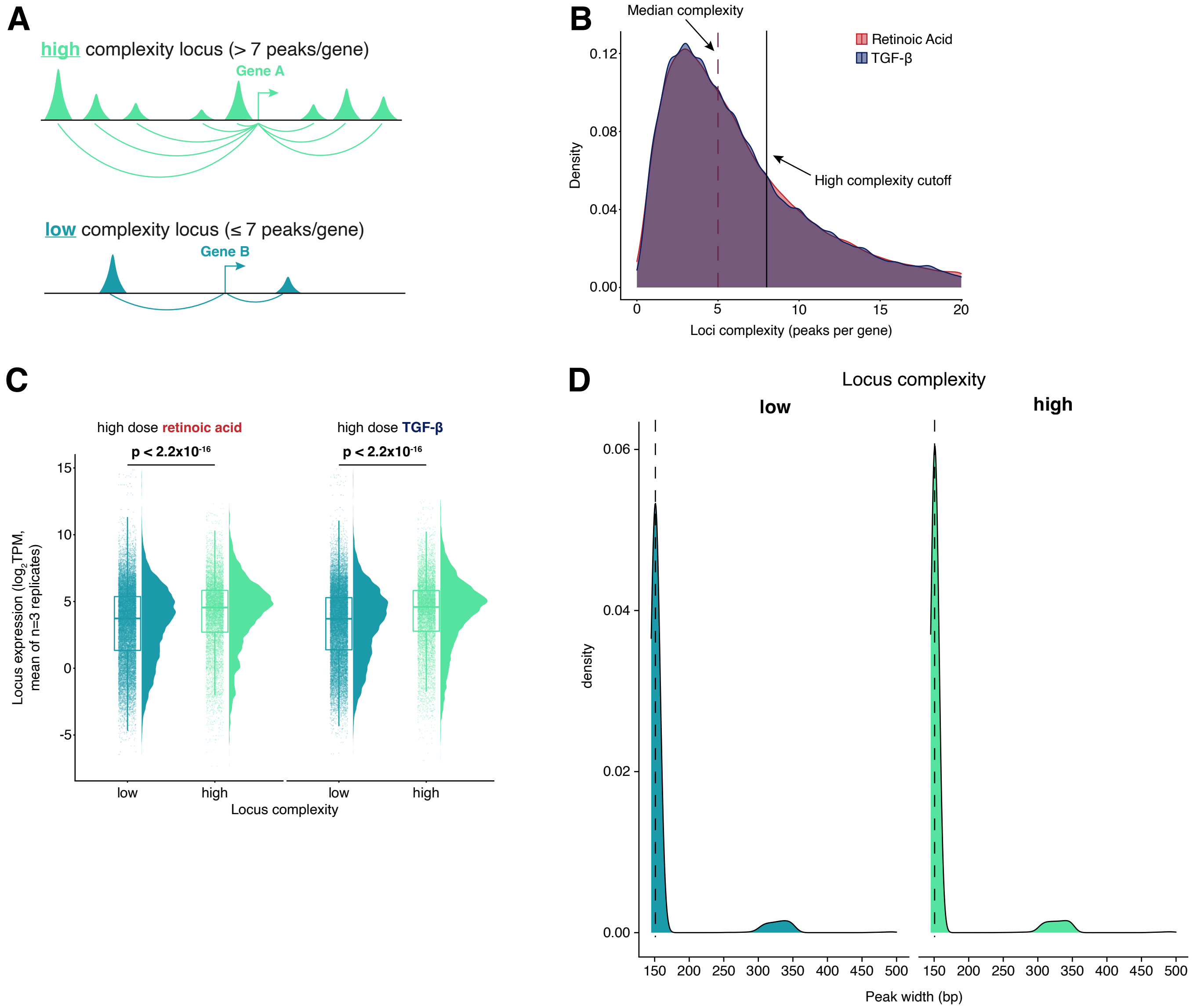

Appendix Figure S7. Expression and peak width distributions in MCF-7 signal data based on locus complexity. (A) Schematic demonstrating classification of genes into "high" versus "low" complexity genes based on the number peaks assigned to a gene using the 'nearest' approach. (B) Density plot of number of peaks per gene in retinoic acid (red) and TGF-β (blue, overlap in purple) with median complexity marked by dotted line and high complexity cutoff marked by solid line. (C) log2-transformed expression of low complexity (teal) and high complexity genes (green) in response to retinoic acid (left) and TGF-β (right). P-values represent the probability of these data or more extreme under the null hypothesis that the distribution of gene expression values were drawn from the same probability distribution via the Kolmogorov-Smirnov test. (D) Distribution of peak widths for low complexity (teal) and high complexity (green) peaks with the median peak width (151 base pairs) marked by the dotted black line.

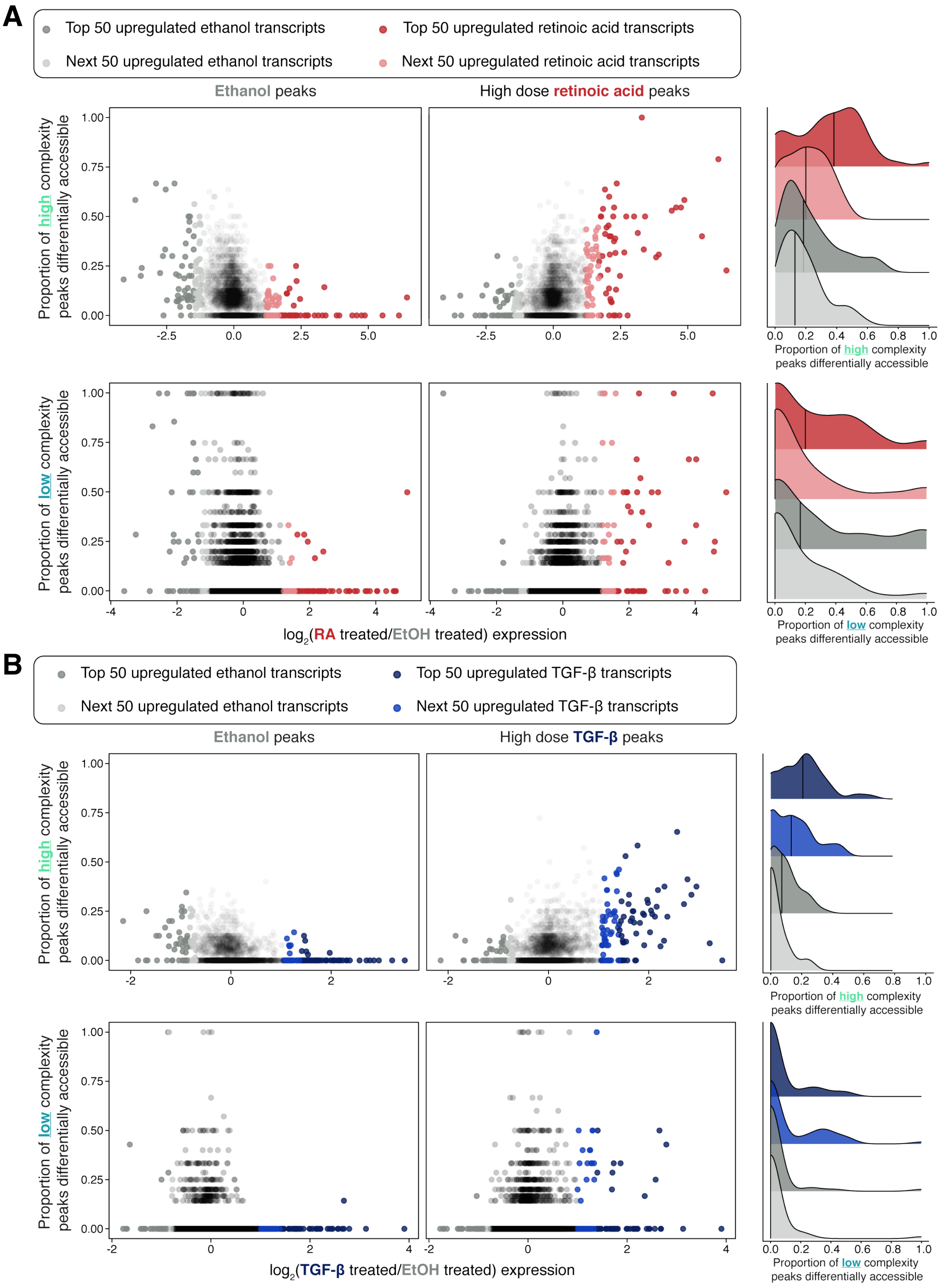

Appendix Figure S8. Concordance between gene expression change and proportion of differentially accessible peaks per gene for high and low complexity genes using a lower minimum coverage threshold for differential peaks. (A) Concordance between expression and accessibility changes between cells exposed to ethanol vehicle control and high dose retinoic acid. Left: plot showing changes in gene expression and chromatin accessibility between ethanol vehicle control and high dose retinoic acid for high and low complexity genes. Each dot is a gene, and on the x axis is the log2 fold change in expression and on the y-axis the proportion of differentially accessible ATAC-seq peaks for each gene. The top 100 most highly expressed genes in ethanol vehicle control and high dose retinoic acid are colored in shades of gray and red, respectively. Right: density plot of the distribution of the proportion of high complexity ATAC-seq peaks associated with the top 100 expressed genes in ethanol vehicle control and high dose retinoic acid with median value marked by vertical black line. (B) Concordance between expression and accessibility changes between cells exposed to ethanol vehicle control and high dose TGF-β. Left: plot showing changes in gene expression and chromatin accessibility between ethanol vehicle control and high dose retinoic acid for high and low complexity genes. Each dot is a gene, and on the x axis is the log2 fold change in expression and on the y-axis the proportion of differentially accessible ATAC-seq peaks for each gene. The top 100 most highly expressed genes in ethanol vehicle control and high dose retinoic acid are colored in shades of gray and blue, respectively. Right: density plot of the distribution of the proportion of high complexity ATAC-seq peaks associated with the top 100 expressed genes in ethanol vehicle control and high dose retinoic acid with median value marked by vertical black line.

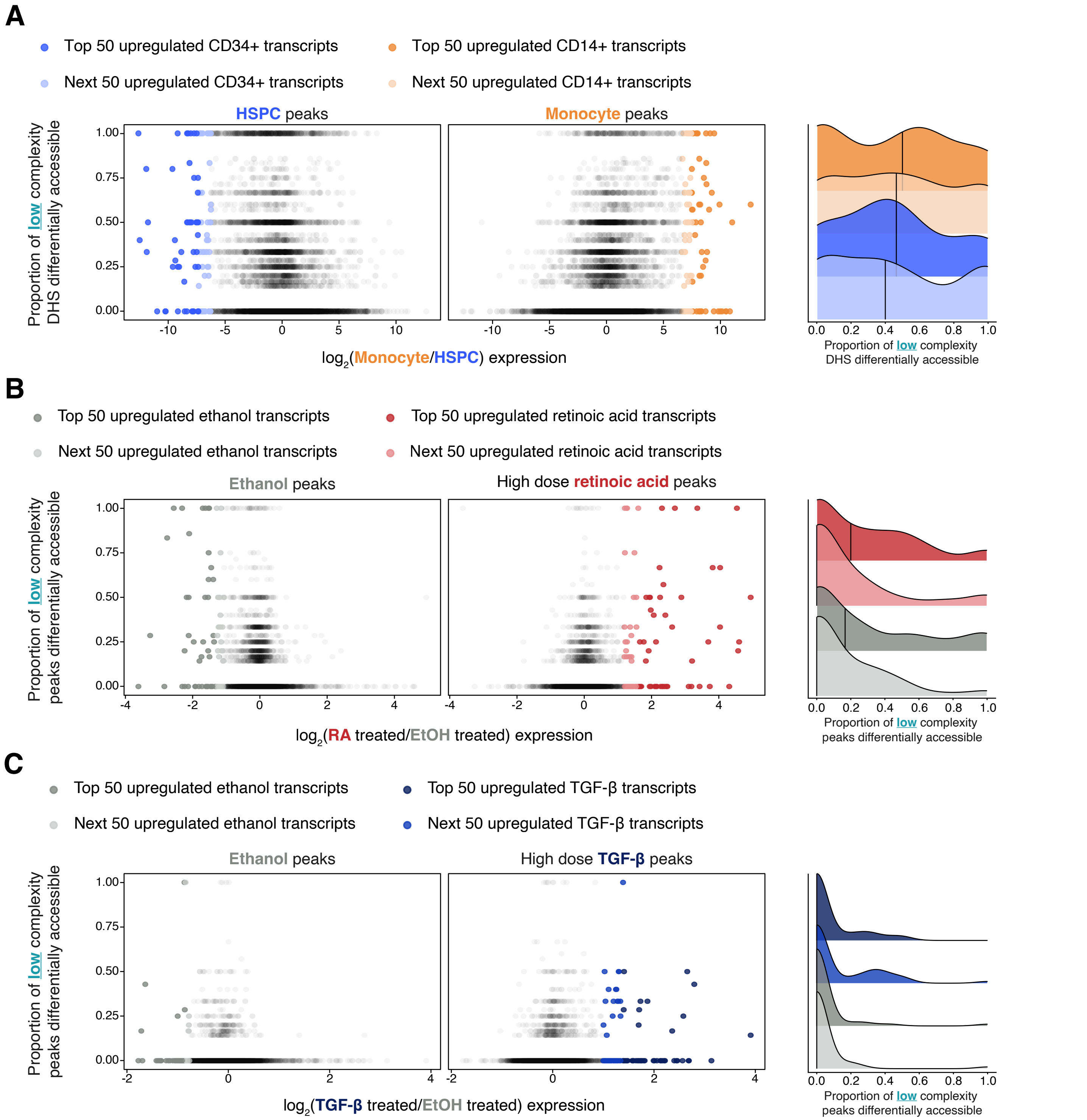

Appendix Figure S9. Concordance between gene expression change and proportion of differentially accessible peaks per gene for low complexity genes. (A) Concordance between expression and accessibility changes between hematopoietic stem and progenitor cells and monocytes. Left: plot showing changes in gene expression in CD34+ hematopoietic stem and progenitor cells (blue) and CD14+ monocytes (orange) from González et al., 2015 (schematic, top). For the plots, each dot is a gene, and on the x axis is log2 fold change in expression and on the y-axis the proportion of differentially accessible DHSs for each associated gene. The top 100 most highly expressed genes in hematopoietic stem and progenitor cells and monocytes are colored in shades of orange and blue, respectively. Right: density plot of the distribution of the proportion of high complexity DHS associated with the top 100 expressed genes in CD34+ hematopoietic stem and progenitor cells and CD14+ monocytes with median value marked by vertical black line. (B) Concordance between expression and accessibility changes between cells exposed to ethanol vehicle control and high dose retinoic acid. Left: plot showing changes in gene expression and chromatin accessibility between ethanol vehicle control and high dose retinoic acid. Each dot is a gene, and on the x axis is the log2 fold change in expression and on the y-axis the proportion of differentially accessible ATAC-seq peaks for each gene. The top 100 most highly expressed genes in ethanol vehicle control and high dose retinoic acid are colored in shades of gray and red, respectively. Right: density plot of the distribution of the proportion of high complexity ATAC-seq peaks associated with the top 100 expressed genes in ethanol vehicle control and high dose retinoic acid with median value marked by vertical black line. (C) Concordance between expression and accessibility changes between cells exposed to ethanol vehicle control and high dose TGF-β. Left: plot showing changes in gene expression and chromatin accessibility between ethanol vehicle control and high dose TGF-β. Each dot is a gene, and on the x axis is the log2 fold change in expression and on the y-axis the proportion of differentially accessible ATAC-seq peaks for each gene. The top 100 most highly expressed genes in ethanol vehicle control and high dose TGF-β are colored in shades of gray and blue, respectively. Righte: density plot of the distribution of the proportion of high complexity ATAC-seq peaks associated with the top 100 expressed genes in ethanol vehicle control and high dose retinoic acid with median value marked by vertical black line.

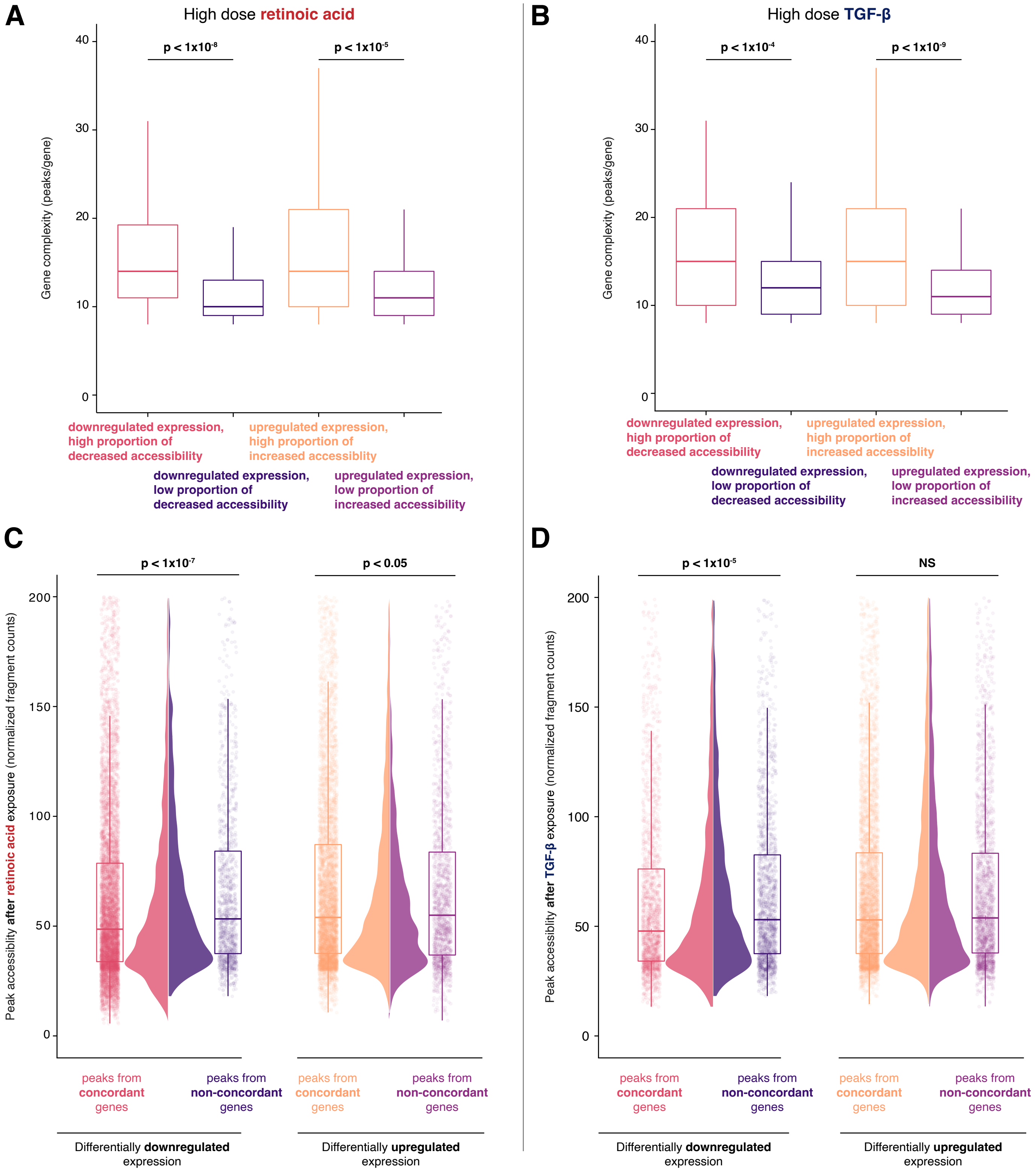

Appendix Figure S10. Accessibility-concordant and accessibility-non-concordant genes have similar loci complexity and differences in peak accessibility after exposure to signal depending on change in gene expression. (A) Distribution of loci complexity the four groups of genes with differential expression in response to high dose retinoic acid. (B) Distribution of loci complexity the four groups of genes with differential expression in response to high dose TGF-β. (C) Accessibility after exposure to high dose retinoic acid. Accessibility of every peak assigned using the 'nearest' approach for gene groups based on accessibility concordance. (D) Accessibility after exposure to high dose TGF-β. Accessibility of every peak assigned using the 'nearest' approach for gene groups based on accessibility concordance. All p-values represent the probability of these data or more extreme under the null hypothesis that the distribution of peak accessibilities were drawn from the same probability distribution via the Kolmogorov-Smirnov test.

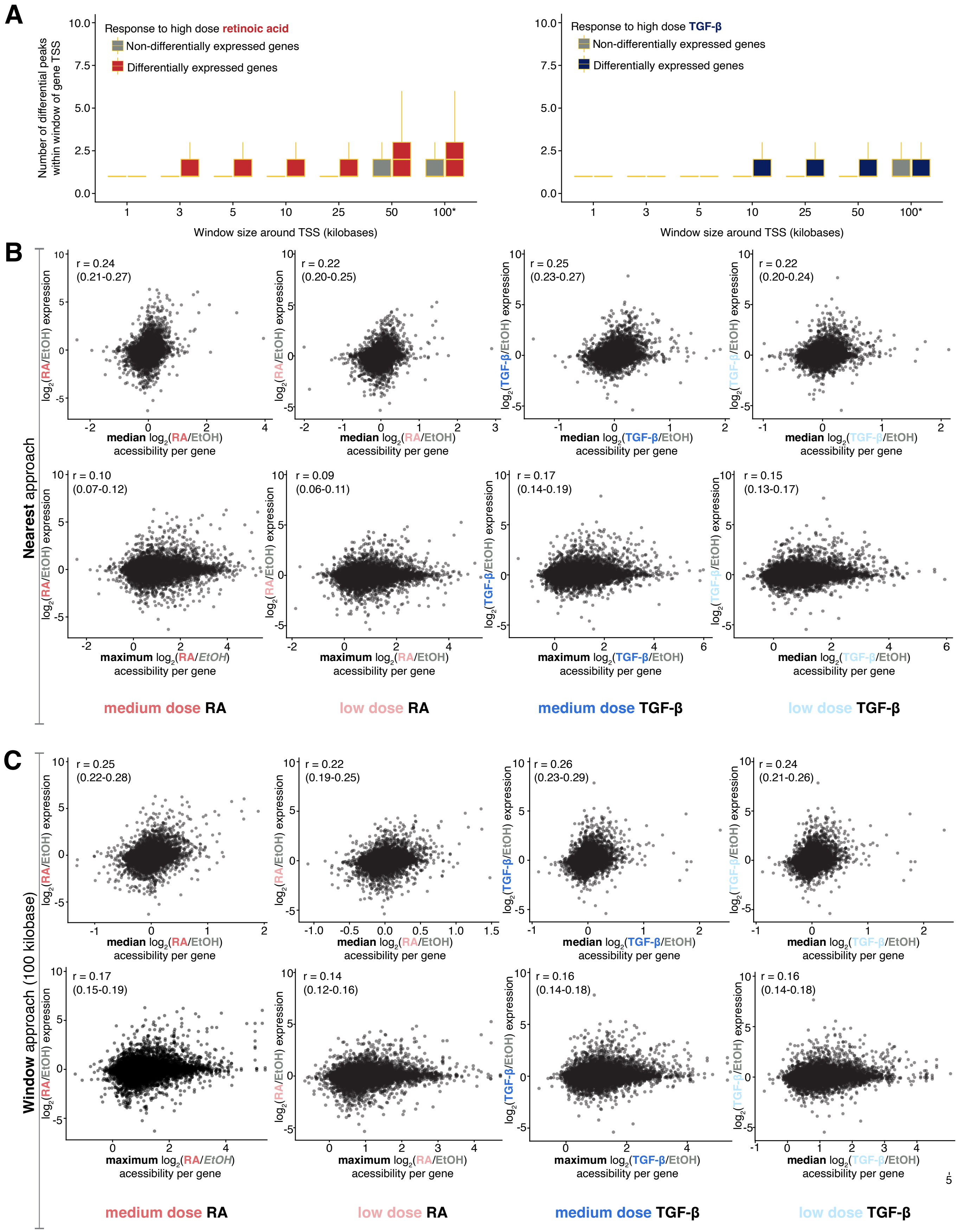

Appendix Figure S11. Effect of window size on number of differentially accessible peaks based on gene expression change and correlation of gene expression and accessibility changes using medium and low dose signals. (A) Distributions of number of differentially accessible peaks for differentially expressed and non-differentially expressed genes in response to high dose retinoic acid (left) or high dose TGF-β (right) based on window size around transcriptional start site (TSS). (B) 'Nearest' approach to assigning peaks to genes shows less concordance in signaling compared to hematopoietic differentiation. Scatter plots showing change in peak accessibility (median or maximum) versus log2 fold change in expression on y axis for medium and low dose retinoic acid (first two columns) and medium and low dose TGF-β (second two columns). Pearson's correlation coefficients reported with 95% confidence interval from bootstrapping with 10,000 replicates in parentheses. (C) 'Window' approach to assigning peaks to genes shows less concordance in signaling compared to hematopoietic differentiation. Scatter plots showing change in peak accessibility (median or maximum) versus log2 fold change in expression on y axis for medium and low dose retinoic acid (first two columns) and medium and low dose TGF-β (second two columns). Pearson's correlation coefficients reported with 95% confidence interval from bootstrapping with 10,000 replicates in parentheses.

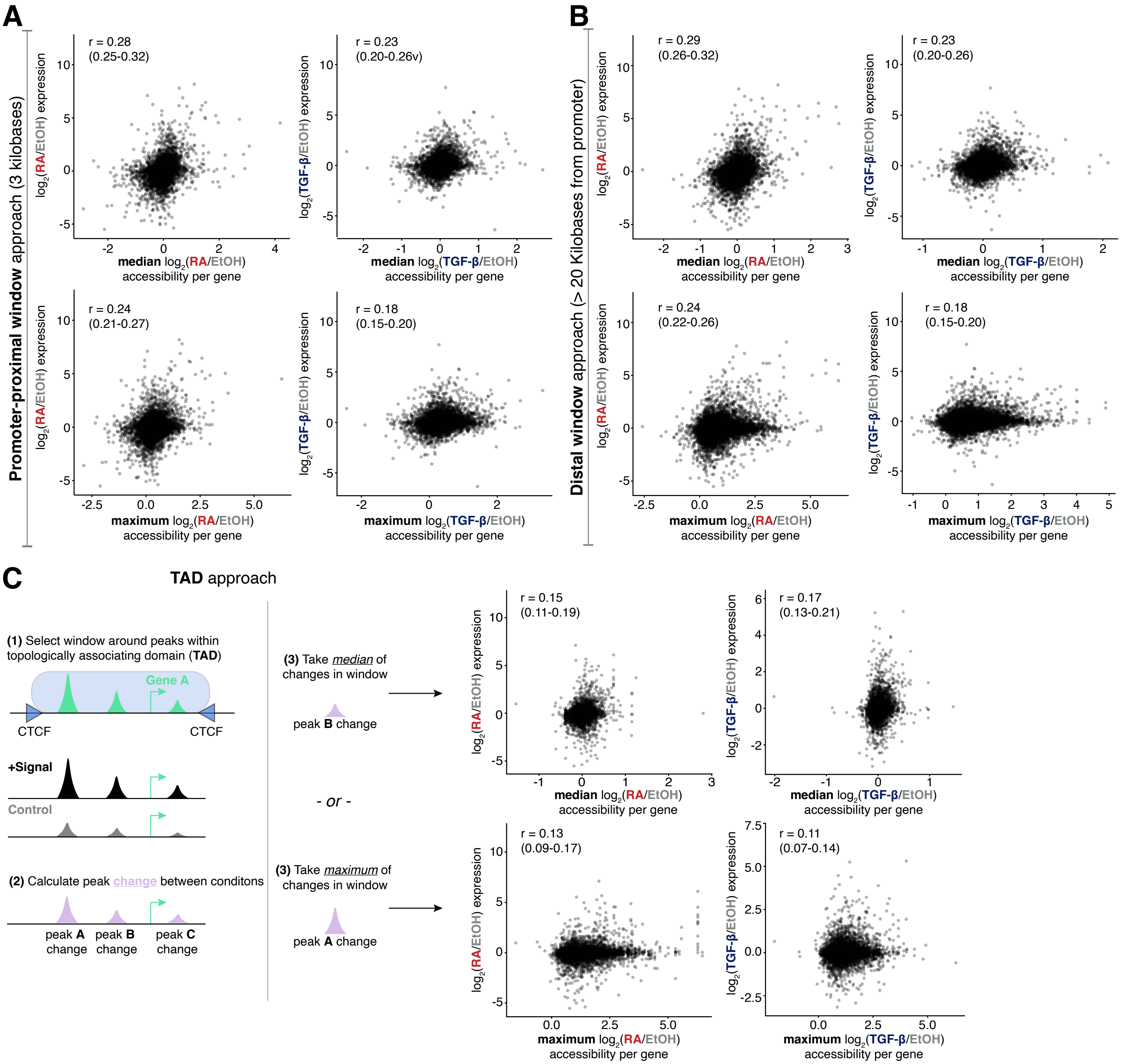

Appendix Figure S12. Alternate window methods for measuring concordance between changes in chromatin accessibility and changes in gene expression. (A) 'Window' approach to assigning peaks to genes using a promoter proximal window (1.5 kilobases up and downstream from TSS). Scatter plots showing change in peak accessibility (median or maximum) versus log2 fold change in expression on y axis for high retinoic acid (first column) and high dose TGF-β (second column). Pearson's correlation coefficients reported with 95% confidence interval from bootstrapping with 10,000 replicates in parentheses. (B) Window' approach to assigning peaks to genes using a promoter distal window (greater than 20 kilobases distal from the promoter). Scatter plots showing changein peak accessibility (median or maximum) versus log2 fold change in expression on y axis for high retinoic acid (first column) and high dose TGF-β (second column). Pearson's correlation coefficients reported with 95% confidence interval from bootstrapping with 10,000 replicates in parentheses. (C) Window' approach to assigning peaks to genes using topologically associating domain (TAD) data to combine peaks from within TADs (schematic). Scatter plots showing change in peak accessibility (median or maximum) versus log2 fold change in expression on y axis for high retinoic acid (first column) and high dose TGF-β (second column). Pearson's correlation coefficients reported with 95% confidence interval from bootstrapping with 10,000 replicates in parentheses.

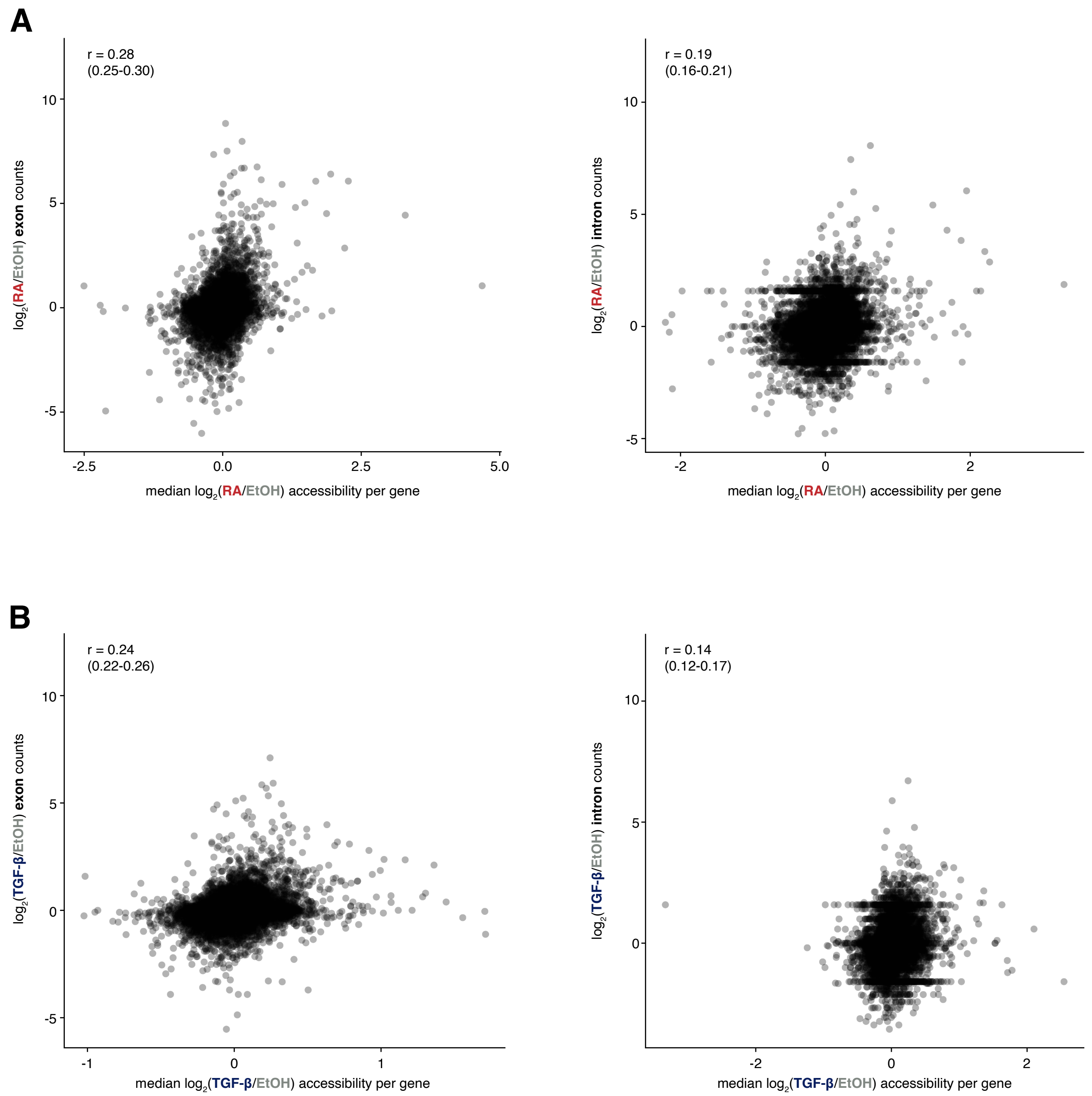

Appendix Figure S13. Subsetting RNA counts based on exonic and intronic reads does not show any further concordance. (A) Using the 'nearest' approach to assigning peaks and splitting RNA reads into exonic (left) and intronic (right) counts for each gene. Scatter plots showing change in peak accessibility (median) versus log2 fold change in expression on y axis for high dose retinoic acid. Pearson's correlation coefficients reported with 95% confidence interval from bootstrapping with 10,000 replicates in parentheses. (B) Using the 'nearest' approach to assigning peaks and splitting RNA reads into exonic (left) and intronic (right) counts for each gene. Scatter plots showing change in peak accessibility (median) versus log2 fold change in expression on y axis for high dose TGF-β. Pearson's correlation coefficients reported with 95% confidence interval from bootstrapping with 10,000 replicates in parentheses.

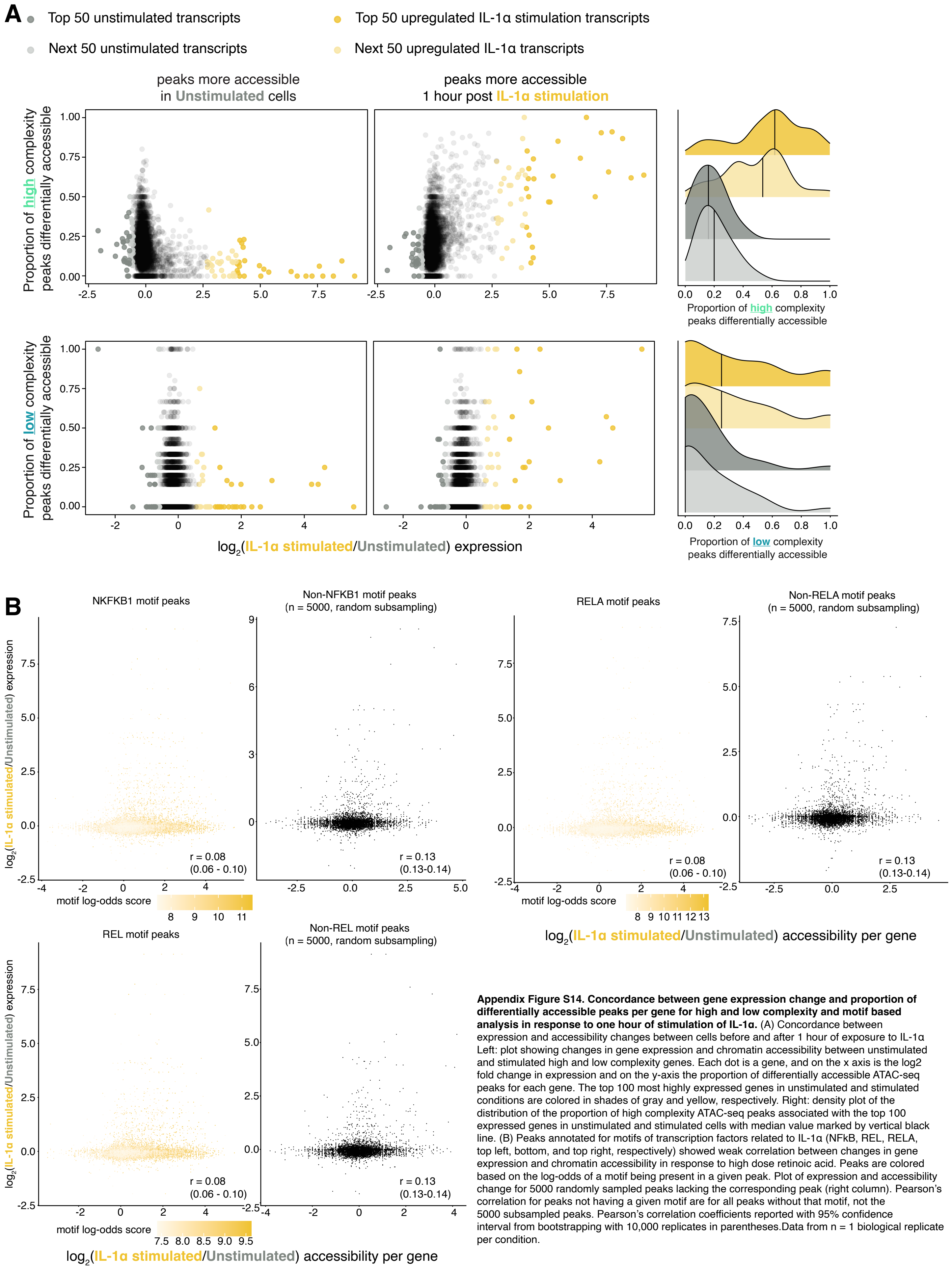

Appendix Figure S14. Concordance between gene expression change and proportion of differentially accessible peaks per gene for high and low complexity and motif based analysis in response to one hour of stimulation of IL-1α. (A) Concordance between expression and accessibility changes between cells before and after 1 hour of exposure to IL-1α Left: plot showing changes in gene expression and chromatin accessibility between unstimulated and stimulated high and low complexity genes. Each dot is a gene, and on the x axis is the log2 fold change in expression and on the y-axis the proportion of differentially accessible ATAC-seq peaks for each gene. The top 100 most highly expressed genes in unstimulated and stimulated conditions are colored in shades of gray and yellow, respectively. Right: density plot of the distribution of the proportion of high complexity ATAC-seq peaks associated with the top 100 expressed genes in unstimulated and stimulated cells with median value marked by vertical black line. (B) Peaks annotated for motifs of transcription factors related to IL-1α (NFkB, REL, RELA, top left, bottom, and top right, respectively) showed weak correlation between changes in gene expression and chromatin accessibility in response to high dose retinoic acid. Peaks are colored based on the log-odds of a motif being present in a given peak. Plot of expression and accessibility change for 5000 randomly sampled peaks lacking the corresponding peak (right column). Pearson's correlation for peaks not having a given motif are for all peaks without that motif, not the 5000 subsampled peaks. Pearson's correlation coefficients reported with 95% confidence interval from bootstrapping with 10,000 replicates in parentheses.Data from n = 1 biological replicate per condition.

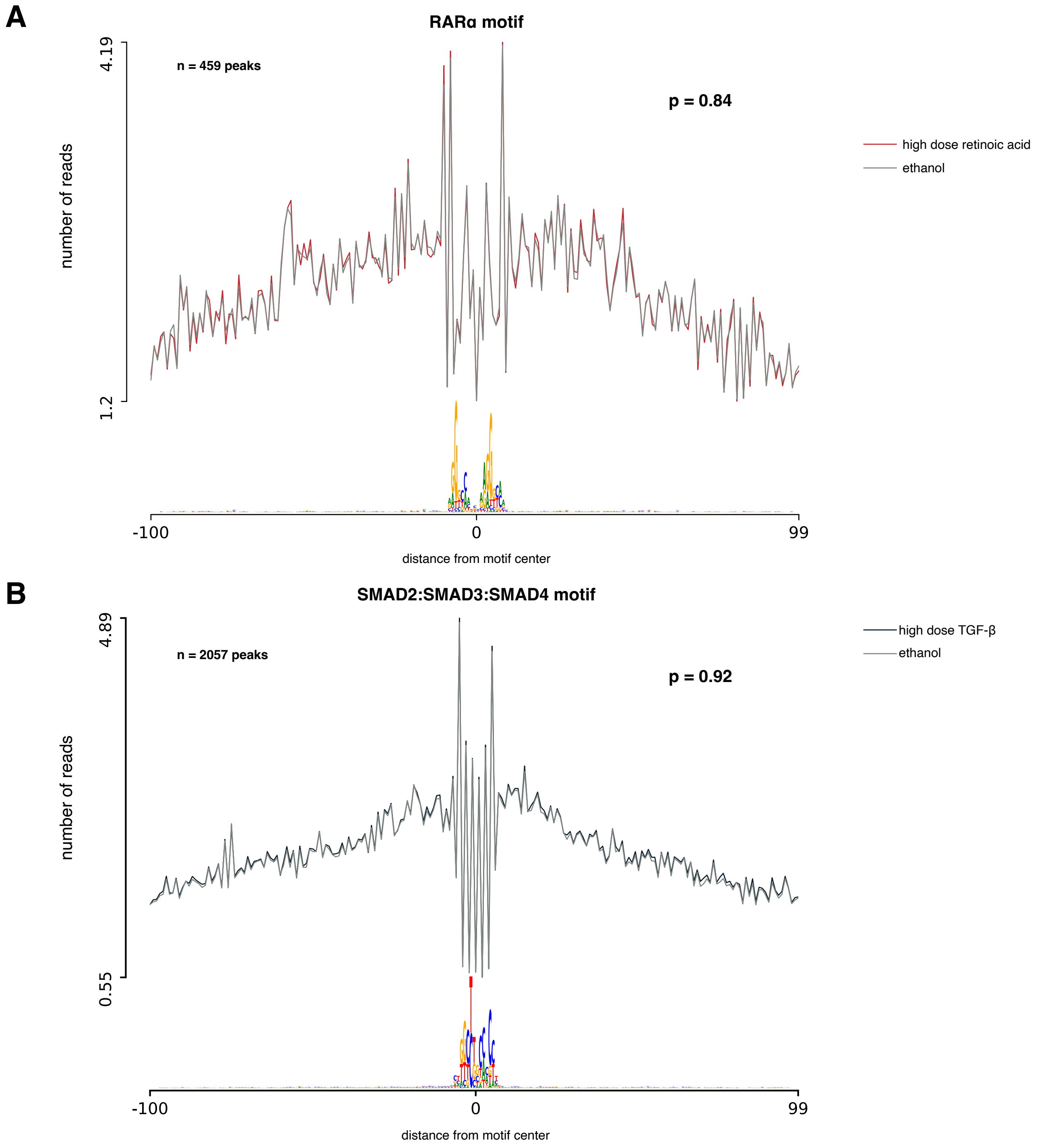

Appendix Figure S15. Subpeak footprinting analysis does not show any changing peaks that can explain concordance Using HINT-ATAC for transcription factor footprinting of non-differentially accessible peaks, we plotted the read density from motif centers for biologically relevant motifs from combined ATAC-seq profiles across n=3 samples. Number of identified peaks shown in top left corner and multiple hypothesis testing-adjusted p-values calculated for the difference between conditions is shown in the top right. (A) Difference between high dose retinoic acid (red) and ethanol control (gray) for retinoic acid receptor alpha motif. (B) Difference between high dose TGF-β (blue) and ethanol control (gray) for combination SMAD motif.

A

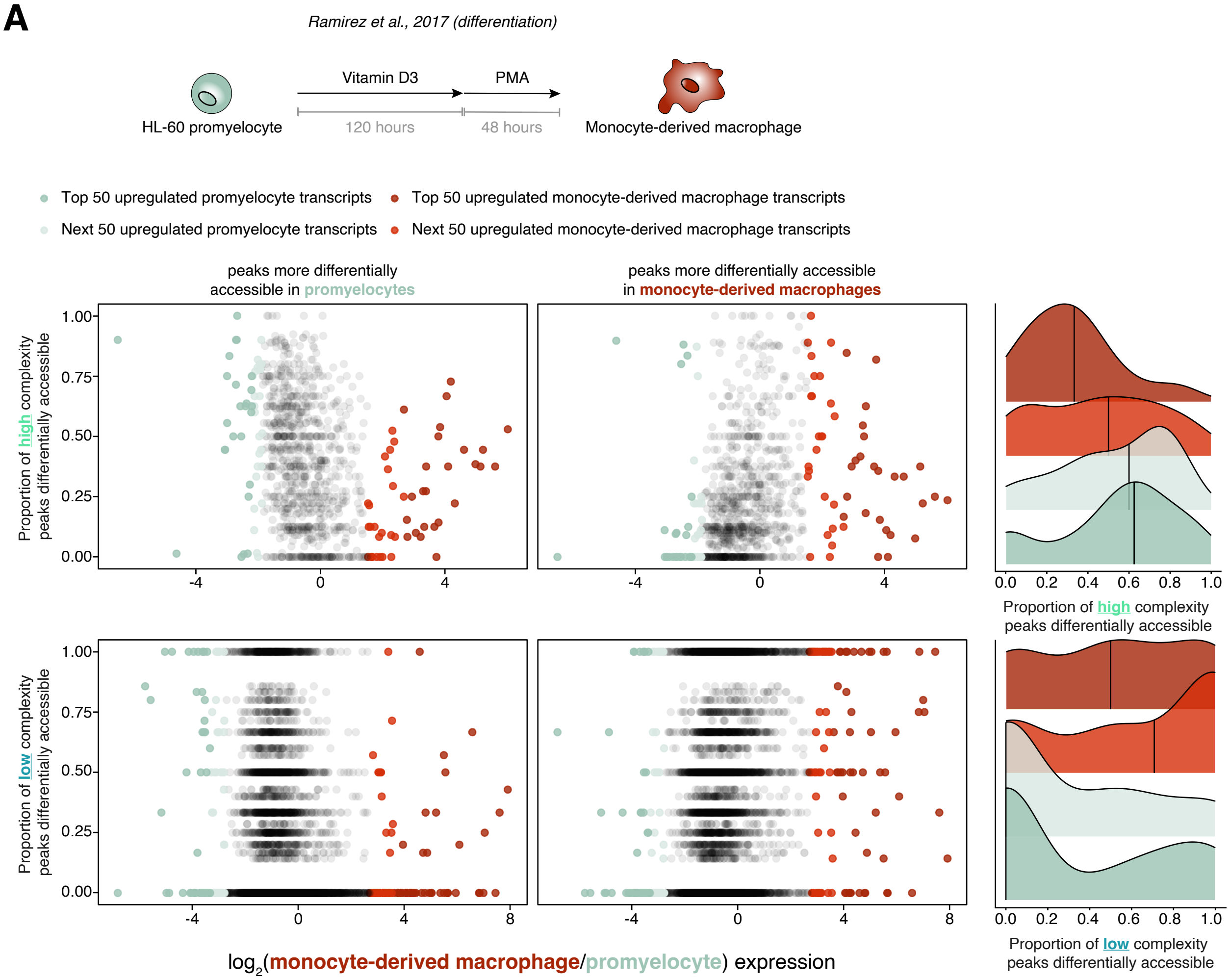

B

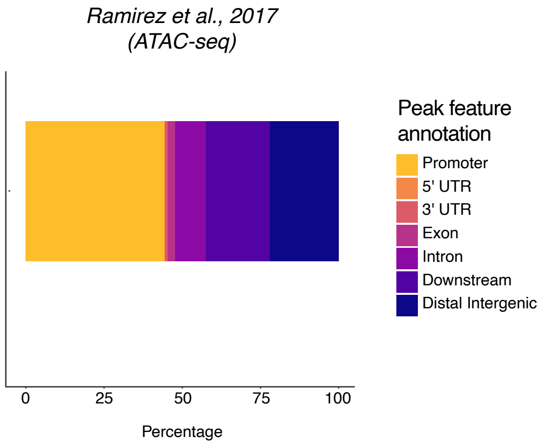

Appendix Figure S16. Concordance between gene expression change and proportion of differentially accessible peaks per gene for high and low complexity genes using a paired RNA-seq and ATAC-seq data set of differentiated promyelocytes from Ramirez et al., 2017. (A) Concordance between expression and accessibility changes between HL-60 promyelocytes before and after a two step differentiation process to turn them into monocyte-derived macrophages (top). Left: plot showing changes in gene expression and chromatin accessibility between HL-60 and monocyte-derived macrophage high and low complexity genes. Each dot is a gene, and on the x axis is the log2 fold change in expression and on the y-axis the proportion of differentially accessible ATAC-seq peaks for each gene. The top 100 most highly expressed genes in HL-60 and monocyte-derived macrophages are colored in shades of green and scarlet, respectively.
Right: density plot of the distribution of the proportion of high complexity ATAC-seq peaks associated with the top 100 expressed genes in HL-60 promyelocytes and monocyte-derived macrophages with median value marked by vertical black line. (B) Annotation of distribution of peak location in relation to gene transcriptional units for consensus files for HL-60 promyelocytes and monocyte-derived macrophages. Data from n = 3 biological replicates per condition.
