# Supplementary material for: Changes in chromatin accessibility are not concordant with transcriptional changes for single‐factor perturbations
Source: Mol Syst Biol. 2022 Sep 7;18(9):e10979. doi: 10.15252/msb.202210979 (PMC9450098; doi:10.15252/msb.202210979)
Supplement: Supplementary file 2 [file MSB-18-e10979-s002.pdf]

Chromatin accessibility changes are not concordant with transcriptional changes for single signals

Karun Kiani, Eric Sanford, Yogesh Goyal, and Arjun Raj
DOI: 10.15252/msb.202210979

Corresponding author(s): Arjun Raj (arjunrajlab@gmail.com)

Review Timeline:

Submission Date:	15th Feb 22
Editorial Decision:	24th Mar 22
Revision Received:	27th Jun 22
Editorial Decision:	22nd Jul 22
Revision Received:	11th Aug 22
Accepted:	19th Aug 22

Editor: Maria Polychronidou

Transaction Report:

(Note: With the exception of the correction of typographical or spelling errors that could be a source of ambiguity, letters and reports are not edited. Depending on transfer agreements, referee reports obtained elsewhere may or may not be included in this compilation. Referee reports are anonymous unless the Referee chooses to sign their reports.)

RE: MSB-2022-10979, Chromatin accessibility changes are not concordant with transcriptional changes for single signals

Thank you again for submitting your work to Molecular Systems Biology. We have now heard back from the three reviewers who agreed to evaluate your study. As you will see below, the reviewers think that the study seems like a relevant contribution to the field. They do however list a series of concerns, which we would ask you to address in a revision.

I think that the reviewers' comments are clear and seem straightforward to address. As such, I do not see the need to repeat any of the points listed below. All issues raised by the reviewers would need to be satisfactorily addressed. Please contact me in case you would like to discuss in further detail any of the issues raised, I would be happy to schedule a call. As you may already know, our editorial policy allows in principle a single round of major revision, so it is essential to provide responses to the reviewers' comments that are as complete as possible.

On a more editorial level, we would ask you to address the following points:

Reviewer #1:

The premise of this manuscript by Kiani et al. is that upon stimulating cells with a given stimulus (in this case TGFbeta or RA), there exists a large numbers of gene loci that respond at the level of transcription, but remain largely unchanged as regards chromatin accessibility at cis-regulatory elements. This is an effect that has of course been noted before in multiple occasions, but the authors try to systematically assess it. The manuscript is well written (perhaps a bit too technical on some aspects, but justifiably so given its goal), figures are of high quality and the analysis reasonable and thorough. However, I feel that there exist aspects of this work that would need to be reworked or put in a different context before the manuscript is considered for publication in MSB. I am listing these below, and I hope that they prove useful for the purposes of revision.

1. The choice of stimulus is most critical here. THe authors have chosen TGFbeta and RA, both of which are "slow" acting stimuli and have, thus, studied changes after 72h. However, there exist reports of acute signaling (e.g., TNFalpha or IL1A) where immediate and possibly more concordant changes occur (e.g., Weiterer et al, EMBO J, 2020) within minutes to few hours. This should be noted here -- and the authors should ideally apply their analysis to an existing such dataset or generate one using an acutely-acting stimulus. I would highly recommmend TNF or IL1A, or at least an early RA time point (earlier than 24 h).

2. Another aspect that is crucial is the nature of the TFs operating downstream each stimulus. As some TFs are more likely to exert some sort of "pioneering" function, they may be more likely to induce larger (and more concordant to gene expression) changes to chromatin accessibility. Again, it seems that NFkappaB that is downstream of TNF/IL1A might do this more efficiently than perhaps SMAD proteins. Thus, "less pioneering" TFs are more likely to use existing accessible chromatin due to their inability to render closed chromatin sites open de novo.

3. Another potentially important aspect is the kind of accessibility changes observed. Despite the authors' nice analysis there is a lack of two things. First, the magnitude of changes expected given the assay used (in our hands ATAC-seq is less sensitive as regards dynamic range, especially at enhancers, comapred to DNase I-seq, and I think that the supplemental analysis here reflects this). Second, it is not only changes in magnitude that are important, but also changes in the patterns of accessibility inside a given ATAC/DHS peak. Using algorithms like HINT (Gusmao et al., Nat Methods 2015), other groups have been able to show that a given accessible peak is actually itself a mini-landscape of less and more accessible sub-regions, and changes to these subregions can very well facilitate or abrogate TF binding. In order for these to be assessed though, enough ATAC/DHS coverage is needed. So, I would urge the authors to try this and, at the very least, comment on whether e.g. SMAD TF motifs become accessible inside of their "unchanging" ATAC peaks upon stimulation.

4. Last, despite the authors' best efforts (they did try different approaches with similar outcomes), it still remains a challenge to assign a given ATAC peak to a gene without some sort of Hi-C/HiChIP data. I would not expect authors to generate such data, but there exist lots of good quality Hi-C data from MCF7 cells and the authors could exploit these. One can simply lift TAD coordinates from such TAD and then confine their connections within the boundaries of TADs to improve calling. We have tested this and adds 10-15% more enhancer assignment to diff-expressed genes.

Minor points:
- Is the RNA-seq analysis capable of also looking at nascent RNA? if this is only mRNA-seq, then sensitivity will be lower as it will mostly reflect changes in steady-state RNA, especially after 72h. We and others have seen that studying nascent RNA (either via a dedicated approach or simply by doing differential analysis of intronic signal from total RNA-seq data) better stratifies activated genes and should affect the analysis done here.
- Please reformat the Supplementary Material to pair figures and figure legends. The current format makes the paper difficult to follow.
- It is not clear to me how the Fischer's exact tests were used to assess if something occurs more than expected by chance. Was it by comparing overlap/fractions in reponsive vs non-responsive genes? I was of the impression that using a hypergeometric test is a better measure for this as it also takes into account that size of the data pool one draws from (of all active genes in this case).
- I might have missed this, but what about genes that are down-regulated and lose accessibility? Were these analysed?
- Did the authors try to separate promoter-proximal from really distal (>20 kbp) ATAC peaks and redo the analysis to see if the relative enrichment changes?

Reviewer #2:

The major goal of this manuscript is to investigate the concordance between gene expression and chromatin accessibility. To do this, the authors analyzed data from MCF-7 cells which were subjected to two different environmental stimulations (retinoic acid and TGF-beta) and paired ATAC-seq/RNA-seq from a hematopoietic different. From these data, the authors observed two classes of genes: 1) genes whose expression changes were accompanied by changes in local chromatin accessibility and, 2) genes which no change in chromatin accessibility could be detected, concluding that gene expression changes can occur irrespective of chromatin accessibility changes.

While the conclusions are not surprising -- TFs that drive chromatin accessibility and not necessarily the drivers of function -- this study represents a strong framework to resolve the qualitative and quantitative relationship between chromatin accessibility and

gene expression. Overall, I have no concern with respect with major conclusions of the manuscript however, I think the presentation (figures) complicate the relatively simple conceptual findings. In particular, I found Fig 2C,D,E (left panels) and Fig 3 (some of the main conclusions of the manuscript) challenging to interpret.

Comments/concerns:

1. Introduction, paragraph 4: "it is unclear how well accessibility-based methods capture activity of all transcription factors. It is possible that some transcription factors' binding and activity does not result in corresponding changes in accessibility and vice-versa." The authors briefly address the previous work studying the binding of TFs with respect to open chromatin (John et al. Nat. Genetics 2011). It has been shown that GR/ER binds almost exclusively to sites with preexisting chromatin accessibly despite considerable changes in gene expression. In a follow-up study from Gordon Hager's group (Biddie et al. Mol. Cell 2011) they show that these sites are bookmarked by AP-1 that maintains accessibility in the absence of an environmental stimulus. It would be useful for the authors to contextualize these previous findings in the introduction. More broadly, a discussion about the environmental stimulation in cell lines vs. cellular differentiation would be helpful.

2. Fig 1C. I find the breakdown of differentially accessibility sites and expression over-simplifies the likely reality of the data. How many ATAC-peaks are assigned to each gene? How many ATAC-seq peaks have reduced accessibly, or conversely increased accessibility. How many genes are associated with two or more ATAC-seq peaks which have divergent changes in accessibility vs. genes with concordant changes in chromatin accessibility? How do you deal with the case where a gene is associated with both up-regulated and down-regulated ATAC/DNase I peaks?

3. Fig 1B; right panel (motifs). The authors should note the motifs shown represent only 4 distinct recognition sequences (RARA, HOX, SMAD and AP1 (TGAgTCA)).

4. Fig 1C. I find the breakdown of differentially accessibility sites and expression over-simplifies the likely reality of the data. How many ATAC-peaks are assigned to each gene? How many ATAC-seq peaks have reduced accessibility, or conversely accessibility. How many genes are associated with two or more ATAC-seq peaks which have divergent changes in accessibility vs. genes with concordant changes in chromatin accessibility?

5. "We observed that approximately 55% of peaks from hematopoietic differentiation data overlapped with peaks from the MCF-7 signal response data". Does the 55% refer to all peaks, irrespective if they are classified as differentially accessible? In any case, I am not convinced that qualitative comparison of the ATAC/DNaseI windowed density at three loci demonstrates that systematic differences are not present. In fact, the authors noted in the previous paragraph (and in Supplementary Fig. 2B) that there exist quantifiable differences in the genomic distribution of differentially accessible peaks between the DNase and ATAC data (possible promoter bias for ATAC). How many total reads map to promoter proximal peaks vs. distal sites in each dataset?

6. Supplementary Fig 5B. It is not possible to distinguish the peak widths of low complexity vs. high complexity (teal vs. green). I suggest a different way to compare these two classes (such as a boxplot).

7. Pg 6. "By comparison, the distribution was skewed toward more (lower complexity) genes having a lower proportion of peaks being differentially accessible in the concordant direction in response to signals in MCF-7 cells, especially in the case of TGF-β". Is this expected, given that the total number of differentially accessible peaks in the hematopoietic differentiation data is ~2X larger than the MCF-7 differential peaks?

8. Fig 3A/B. From my understanding of the figure legend the y-axes refer to the proportion of ATAC/DNase I peaks that have concordant changes (i.e., gene expression increases, ATAC/DNAse I peak increases). The value that distinguishes low proportion of concordant accessibility vs. changes to its peaks appears to be 10% (dotted line in figure panel). What is the rationale behind this threshold value?

9. Supplementary Fig 4. panels are not labelled.

Reviewer #3:

This manuscript by Kiani et al utilizes previously published paired chromatin accessibility and RNA expression datasets to address the question of the association between changes in RNA expression and chromatin accessibility. Using these datasets they conclude that many changes in RNA expression are not associated with changes in chromatin accessibility, a finding that is more evident with their single-factor perturbation data as opposed to the cellular differentiation datasets. In addition, they categorize this finding based on genes with more neighboring regulatory elements. Overall, the conclusions drawn by the authors are not entirely surprising based on prior studies that have looked into this finding. However, their conclusion regarding the differences between single-factor perturbation data as opposed to the cellular differentiation datasets is of interest. Below are some comments.

The abstract makes a statement regarding a causal relationship between changes in accessibility and changes in expression. Specifically, it says that "some changes to accessibility changes may occur after changes to expression". As the datasets that they are evaluating are only for two time points (i.e., before and after treatment), it is quite challenging to ascribe causality. Also, I am unsure where in the manuscript that this conclusion is being derived from.

The authors should spend more time discussing the John et al., paper (PMID 21258342) and Samstein et al paper (PMID 23021222) in the introduction. These papers serve as a foundation in terms of how single-factor perturbations and cellular differentiation impact chromatin accessibility profiles. A lot of the findings regarding chromatin accessibility that the authors present in this manuscript are documented in these prior studies.

The structure of this manuscript could be significantly improved. It felt as though it was jumping around a lot, and was quite verbose for what in the end is a rather simple conclusion.

Figure 1E. Can you please show the mapped RNA reads on this browser figure. It is unclear where these RNA reads are coming from, as the transcriptional start site in the annotated gene does not appear to be accessible in the provided plot.

Figure 1C. Please correct the Venn diagrams to make the individual circles appropriately scaled.

The data presented in Sup Figure 8 does not appear to support the conclusion "We quantified the distribution of peak complexity across these groups and observed that they were similar across all four gene subgroups (Supplemental Figures 8A,B)". Specifically, it appears that the "upregulated expression, high proportion of increased accessibility" has significantly higher gene complexity than the "upregulated expression, low proportion of increased accessibility". Please provide p-values for the comparisons on this plot and correct this statement accordingly.

The authors mention differences in the distribution of promoter versus distal peaks in the ATAC-seq and DNAseI-seq datasets that they are using. ATAC-seq has a known bias towards promoters, so this is not surprising. The authors try to correct for this in Figure 4C, but they should further comment on whether this difference could explain the difference they are seeing between the single-factor perturbation data as opposed to the cellular differentiation datasets? Are there differentiation datasets they can use that are also ATAC-seq?

Reviewer #1:

The premise of this manuscript by Kiani et al. is that upon stimulating cells with a given stimulus (in this case TGFbeta or RA), there exists a large numbers of gene loci that respond at the level of transcription, but remain largely unchanged as regards chromatin accessibility at cis-regulatory elements. This is an effect that has of course been noted before in multiple occasions, but the authors try to systematically assess it. The manuscript is well written (perhaps a bit too technical on some aspects, but justifiably so given its goal), figures are of high quality and the analysis reasonable and thorough. However, I feel that there exist aspects of this work that would need to be reworked or put in a different context before the manuscript is considered for publication in MSB. I am listing these below, and I hope that they prove useful for the purposes of revision.

We thank the reviewer for their kind assessment of our work and their careful reading of our manuscript. We appreciate the helpful comments and feel as those addressing them has made the manuscript considerably stronger.

1. The choice of stimulus is most critical here. The authors have chosen TGFbeta and RA, both of which are "slow" acting stimuli and have, thus, studied changes after 72h. However, there exist reports of acute signaling (e.g., TNFalpha or IL1A) where immediate and possibly more concordant changes occur (e.g., Weiterer et al, EMBO J, 2020) within minutes to few hours. This should be noted here -- and the authors should ideally apply their analysis to an existing such dataset or generate one using an acutely-acting stimulus. I would highly recommend TNF or IL1A, or at least an early RA time point (earlier than 24 h).

We thank the reviewer for pointing out this critical point about how the choice of stimulus may lead to opposite conclusions. As per their suggestion, we used the RNA-seq data and ATAC-seq data from Jurida et al., 2015 and Weiterer et al., 2020 which look at KB epithelial carcinoma cells before and after 1 hour of stimulation with IL1α. As the reviewer suggested, this acute signaling paradigm demonstrated more concordant changes especially at high complexity loci (see below). We have included this supplementary figure and have altered our text to reflect this possibility.

Discussion:
We looked at MCF-7 cells exposed to retinoic acid and TGF-β because these two signals induce a robust transcriptional response through distinct mechanisms. RAR-α remains bound to DNA and interacts with transcriptional activators in response to retinoic acid binding, while SMAD family members require TGF-β to bind to surface receptors to translocate to the nucleus. Yet, despite these differences, we observed that many genes changed expression independent of changes in chromatin accessibility for both signals. It is, however, possible that signaling molecules that exert their effects through very different types of transcription factors may have a different profile of concordance between changes in accessibility and gene expression. It is possible that other types of factors in a different context (e.g., different cell line) may yield a stronger correspondence. Indeed, some acute stimuli can lead to more concordance (Supplemental Figure 16A). Potential reasons for this difference is the ability of transcription factors such as NFκB to rapidly decondense heterochromatin to quickly mediate inflammatory responses (Weiterer et al. 2020). Further, systematic studies of a number of signaling pathways and timescales will be required to make a complete determination of the degree of concordance in various contexts.

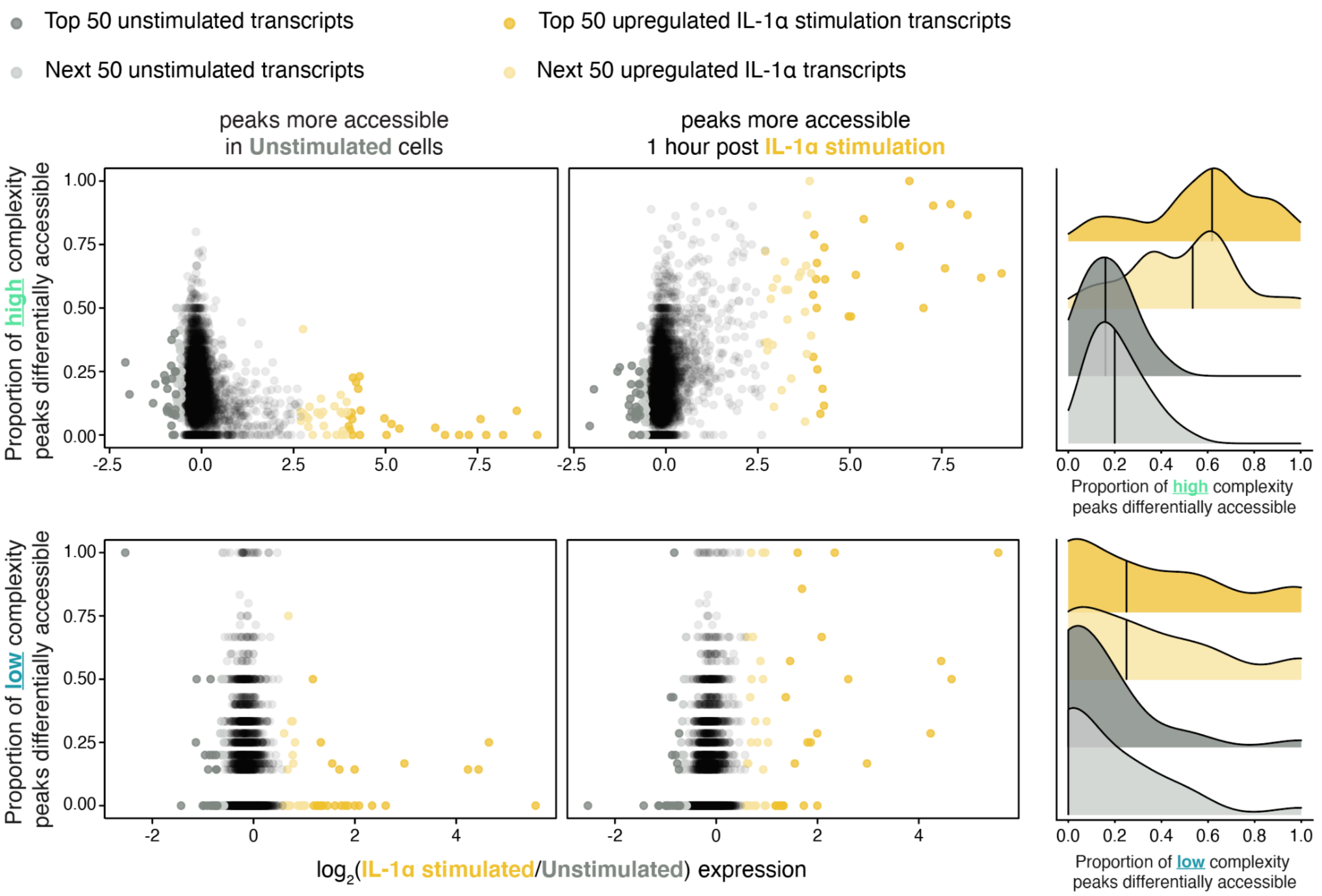

2. Another aspect that is crucial is the nature of the TFs operating downstream each stimulus. As some TFs are more likely to exert some sort of "pioneering" function, they may be more likely to induce larger (and more concordant to gene expression) changes to chromatin accessibility. Again, it seems that NFkappaB that is downstream of TNF/IL1A might do this more efficiently than perhaps SMAD proteins. Thus, "less pioneering" TFs are more likely to use existing accessible chromatin due to their inability to render closed chromatin sites open de novo.

We thank the reviewer for pointing out this important distinction regarding factors that may have more pioneer activity. Moreover, FOXA1, one of the transcription factors we looked at for our retinoic acid data, has been shown to have pioneering activity (Zaret and Carroll, 2011) though it is unclear whether that activity is relevant to our MCF-7 system. Using the previously mentioned data on KB cells before or after one hour of IL1α stimulation, we used the same motif analysis as previously described using three motifs associated with NFκB: NFκB1, REL, and RELA, and were similarly unable to discern any stronger signal when subsetting for these peaks specifically. We have altered our text to describe this new analysis:

> Finally, we wondered if peaks that contained the motifs of transcription factors that are associated with retinoic acid and TGF-β signaling only (as opposed to all peaks) would show a stronger correlation between the changes in chromatin accessibility and gene expression. We annotated peaks with a log-likelihood score of a given motif being found in that peak and subsetted on those peaks with a nonzero log-likelihood score to examine the correlation between changes in accessibility and gene expression. Using this approach, we examined log-likelihood scores for motifs associated with retinoic acid signaling (RARA-α, HOXA13, and FOXA1) and motifs associated with TGF-β (SMAD3, SMAD4, and SMAD9). We observed that focusing on peaks annotated with peaks we would *a priori* expect to be involved in modulating gene expression in response to signal showed limited correlation between changes in chromatin accessibility and changes in gene expression (Figure 5). We

also used this approach to look for peak-expression correlations for transcription factors downstream of IL-1α (NFκB1, REL, and RELA, of which NFκB1 has pioneer activity) and similarly found little correlation between chromatin accessibility and gene expression changes (Supplemental Figure 16B). These results suggest that particular transcription factors show no more concordance between peak changes and expression changes, even for pioneer transcription factors.

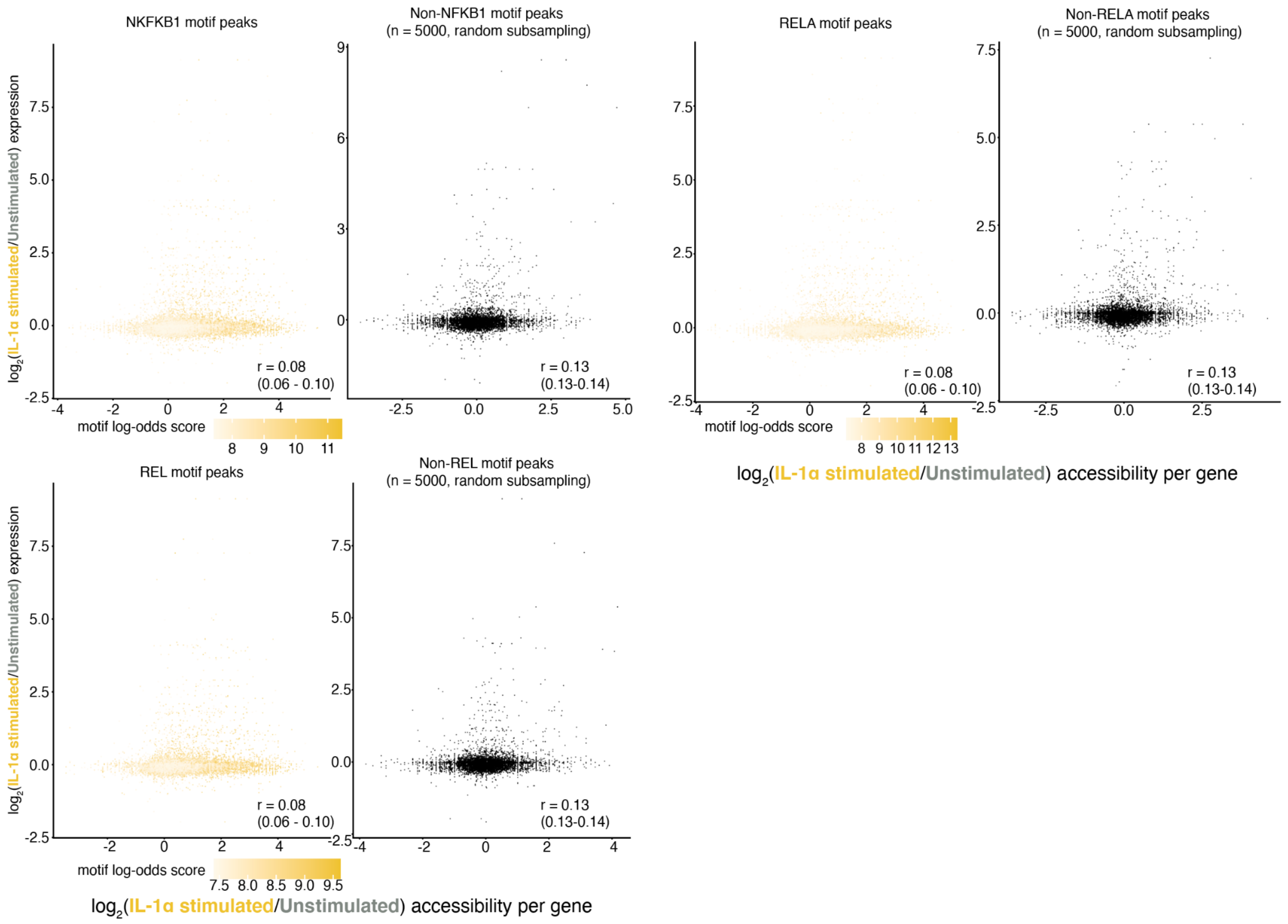

3. Another potentially important aspect is the kind of accessibility changes observed. Despite the authors' nice analysis there is a lack of two things. First, the magnitude of changes expected given the assay used (in our hands ATAC-seq is less sensitive as regards dynamic range, especially at enhancers, comapred to DNase I-seq, and I think that the supplemental analysis here reflects this). Second, it is not only changes in magnitude that are important, but also changes in the patterns of accessibility inside a given ATAC/DHS peak. Using algorithms like HINT (Gusmao et al., Nat Methods 2015), other groups have been able to show that a given accessible peak is actually itself a mini-landscape of less and more accessible sub-regions, and changes to these subregions can very well facilitate or abrogate TF binding. In order for these to be assessed though, enough ATAC/DHS coverage is needed. So, I would urge the authors to try this and, at the very least, comment on whether e.g. SMAD TF motifs become accessible inside of their "unchanging" ATAC peaks upon stimulation.

We thank the reviewer for suggesting HINT as an orthogonal method to try. In order to explore any sub-peaks that may be present in our data, we first subsetted only on those peaks which were not differentially accessible upon exposure to high dose retinoic acid or TGF-β. We then used these peaks as a basis to narrow down the search of the HINT (Zhijian et al., 2019, PMID: 30808370). We

merged the aligned .bam files across all three replicates so the traces represent motif read density across all three replicates. This indicates that within the peaks that are not differentially accessible between conditions, there is no real difference in read density of motifs relevant to transcription factors of interest to retinoic acid or TGF-β biology. Thus it is unlikely that subpeaks within unchanging peaks are facilitating transcription factor binding in our system. We have added this analysis to the supplement and the accompanying text from the results (below) and methods.

> To exclude the possibility that 'subregions' within unchanging peaks could be facilitating transcription factor binding, we measured RARα and SMAD footprints within these peaks.The number of reads between control and exposure conditions did not change for these footprints, indicating that there are no more accessible subregions that could mediate transcription factor binding within peaks that were not differentially accessible (Supplemental Figures 11A,B).

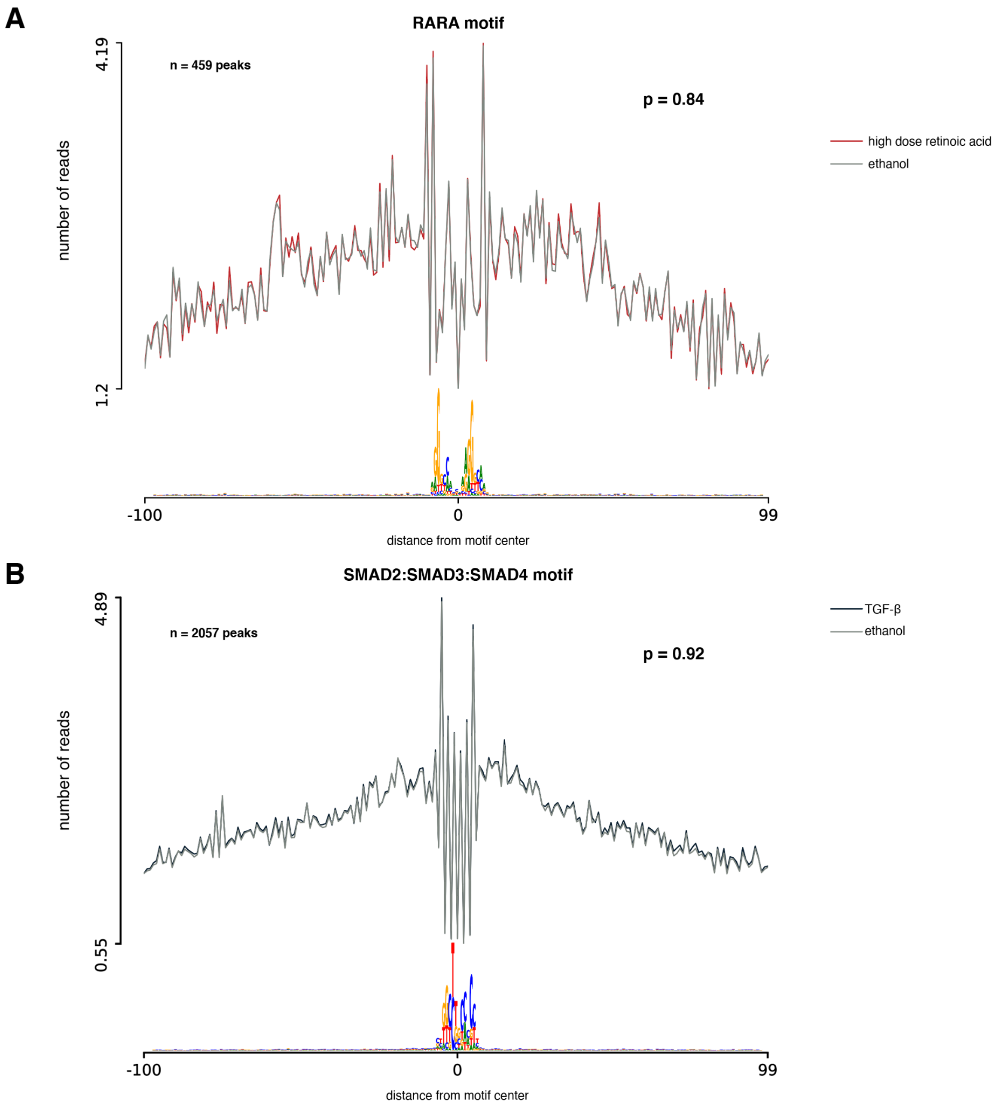

4. Last, despite the authors' best efforts (they did try different approaches with similar outcomes), it still remains a challenge to assign a given ATAC peak to a gene without some sort of Hi-C/HiChIP data. I would not expect authors to generate such data, but there exist lots of good quality Hi-C data from MCF7 cells and the authors could exploit these. One can simply lift TAD coordinates from such TAD and then confine their connections within the boundaries of TADs to improve calling. We have tested this and adds 10-15% more enhancer assignment to diff-expressed genes.

We thank the reviewer for their assessment of our approach and the suggestion to use TAD coordinates for this analysis. We used MCF-7 TAD coordinates from Barutcu et al., 2015, PMID: 26415882 to focus our analysis on a TAD-by-TAD basis. In our case, looking at either the median or maximum of all peaks within the same TAD as the transcriptional start site of the gene of interest did not offer any stronger qualitative or quantitative relationship compared to our 'nearest' and 'window' approaches. We have added these analyses to Supplemental Figure 14C, and have added text discussing this point as follows:

> Next, we used a window-based approach where there was the possibility of a many-to-one mapping of peaks to genes. We assigned all peaks within a 100 kilobase window (Sanford *et al*, 2020) in order to maximize the number of differential peaks assigned to a gene (Supplemental Figure 9A,B). Similar to the 'nearest' approach, we collapsed values using median accessibility change across all peaks assigned to a gene as well as maximum accessibility per gene (Figure 4B, schematic) We observed a similar effect using this approach where there was a stronger correlation between change in accessibility and change in expression between HSPC versus monocyte versus MCF-7 cells exposed to signal (Figure 4B). Of note, the correlation coefficients were similar between both methods of assigning peaks. Additionally, we used the window based method to subset promoter-proximal peaks (i.e, within 1.5 kilobase pairs up or downstream from the transcription site) as well as distal peaks (greater than 20 kilobase pairs from the transcriptional site). This approach similarly did not demonstrate a strong relationship at the concordance between chromatin accessibility changes and gene expression changes in response to retinoic acid or TGF-β (Supplemental Figures 16A,B). We also used a variable window approach by restricting our analyses to all peaks within the same topologically associating domain (TAD), which also did not demonstrate a strong correlation between changes in accessibility and changes in gene expression (Supplemental Figure 16C).

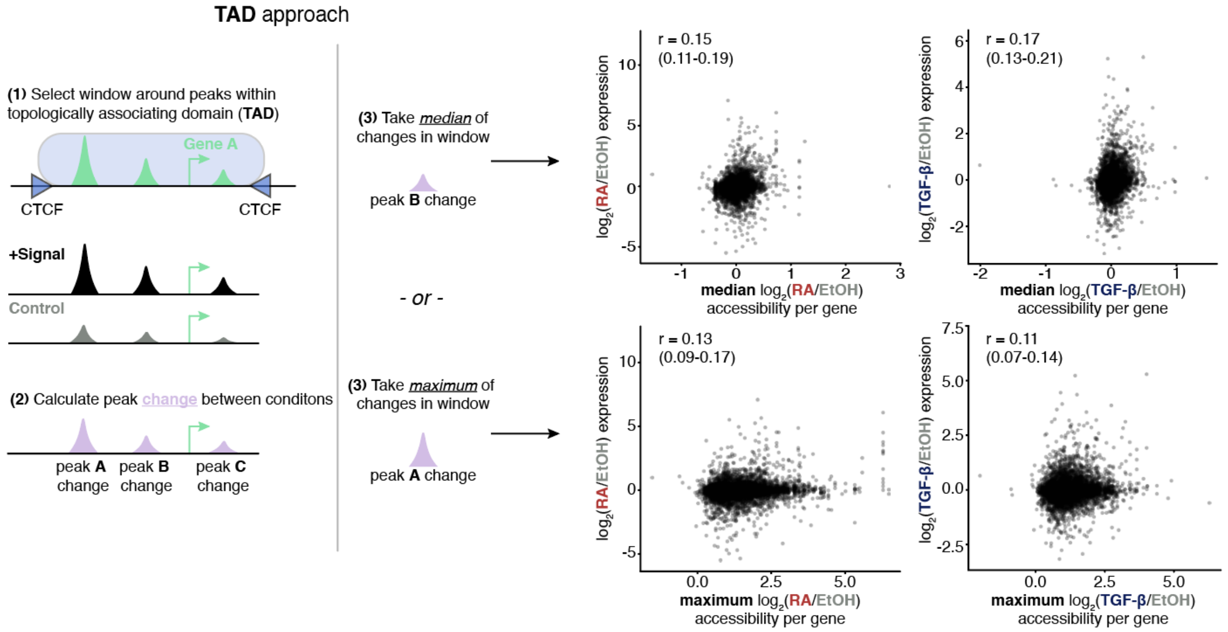

Minor points:

- Is the RNA-seq analysis capable of also looking at nascent RNA? if this is only mRNA-seq, then sensitivity will be lower as it will mostly reflect changes in steady-state RNA, especially after 72h. We and others have seen that studying nascent RNA (either via a dedicated approach or simply by doing differential analysis of intronic signal from total RNA-seq data) better stratifies activated genes and should affect the analysis done here.

We thank the reviewer for the suggestion and agree that nascent RNA may have more concordance. While these RNA data are not as well-situated for measuring nascent RNA as a technique such as global run on sequencing (GRO-seq), we quantified the number of counts from our data mapping to a standard exonic GTF file or to do an intronic GTF file to quantify exon and intron counts on a gene by gene basis. These raw counts were quantile normalized across samples and we used a scatter plot analysis comparing the change in normalized counts between conditions and the median change in accessibility for a given gene, with peaks assigned using the 'nearest method'. The results of this analysis are in Supplemental Figures 15A,B and are discussed in the text below added to the manuscript.

> To see if peaks in specific genomic regions (promoters, parts of the gene body, downstream and intergenic areas) had unique relationships between change in chromatin accessibility and change in gene expression, we subsetted our correlation analysis. We annotated peaks using ChiPseeker (Yu *et al*, 2015) to categorize them as being at promoters, within the gene body (5' UTR, 3' UTR, intronic, and exonic sequences), downstream of the gene end, or at intergenic sequences. We used peaks assigned to genes using the 'nearest' approach and took the median change in accessibility per gene. The strongest correlation between changes in accessibility and gene expression across sets of comparisons was at promoter peaks (Figure 4C). While promoter correlation is quantitatively stronger, the overall qualitative conclusion remains the same. We also quantified changes in intronic reads and compared it to changes in exonic reads in order to determine if there is a stronger relationship between more nascent RNA changes and accessibility changes. However, the quantitative relationship was no better than those using other methods (Supplemental Figure 15). Thus, despite using a variety of approaches for both assigning peaks to genes as well as collapsing the accessibility of all peaks for a given gene to a single value, we failed to appreciate a strong relationship between changes in accessibility and changes in gene expression.

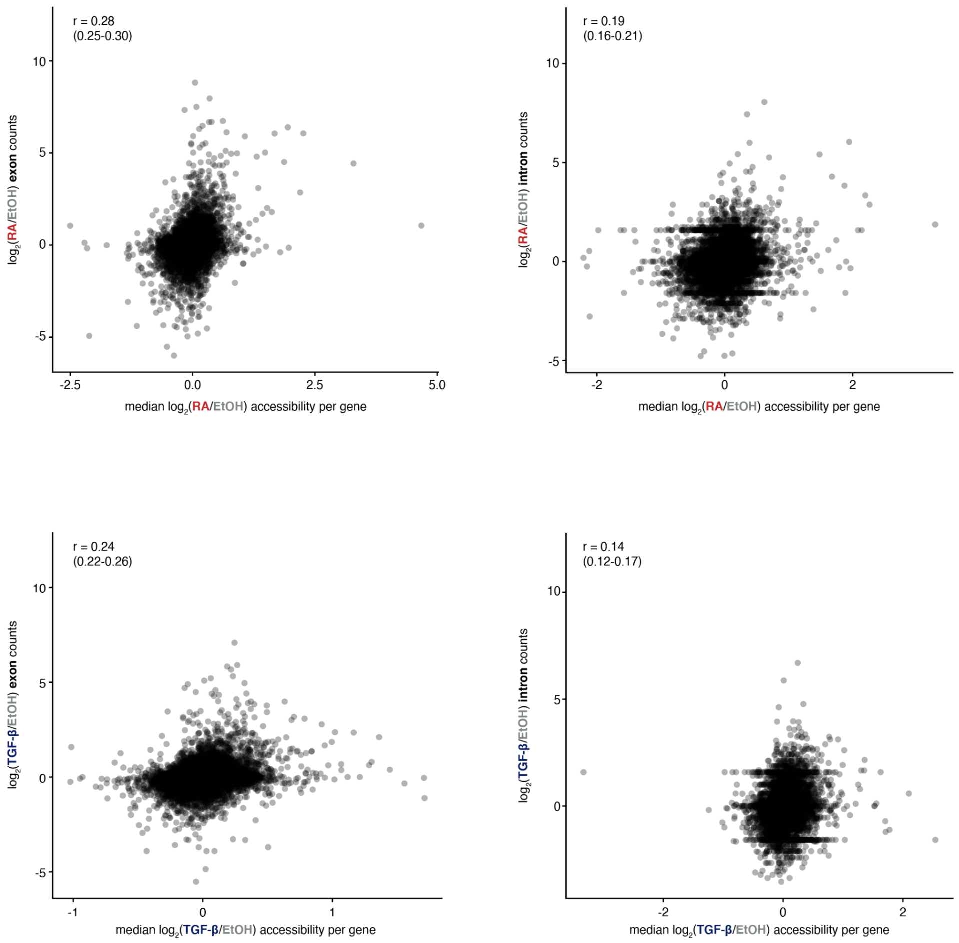

- Please reformat the Supplementary Material to pair figures and figure legends. The current format makes the paper difficult to follow.

We apologize for any lack of clarity—we have reformatted the paper per journal guidelines.

- It is not clear to me how the Fischer's exact tests were used to assess if something occurs more than expected by chance. Was it by comparing overlap/fractions in reponsive vs non-responsive genes? I was of the impression that using a hypergeometric test is a better measure for this as it also takes into account that size of the data pool one draws from (of all active genes in this case).

We thank the reviewer for looking into the statistical details. After consultation, it appears that the Fisher exact test does indeed take into account the size of the data pool, and uses the hypergeometric distribution in its calculation. The test looks at a contingency table of whether or not a given gene has differential expression and whether or not the gene has differential accessibility (in this case if *any* peak assigned to the gene using the 'nearest' method is differentially accessible) in response to retinoic acid or TGF-β. It then compares the contingency table of the data to all possible contingency tables given the same marginal totals (i.e. genes with expression changing/not changing, genes with accessibility changing/not changes) and states how extreme our data is. We have also clarified the statistical procedures in the legend of Figure 1C as follows:

Overlap between changes in gene expression and changes in chromatin accessibility in response to high dose retinoic acid (top) or high dose TGF-β (bottom). Of the genes that were differentially expressed (right circle of Venn diagram) we looked at the overlap (shaded) of how many of the genes also had at least one differentially accessible peak assigned to it using the 'nearest' approach. (left circle). We performed Fisher's exact test to

show the probability of the joint values of genes with overlapping changes in expression and chromatin accessibility in our data compared to all possible combinations.

- I might have missed this, but what about genes that are down-regulated and lose accessibility? Were these analysed?

We apologize for not making this point more clearly. These genes are addressed in the left panel of the plots in what is now Figures 2B-D. In order to better express this point, we have included a schematic in Figure 2A (shown below) to describe how these plots are made and have used example “Gene B” to illustrate a gene with a decrease in expression and decrease in chromatin accessibility in response to a signal. We also analyze down-regulated genes that lose accessibility in Figure 3 and have changed the labeling to make the descriptions more clear. In panels C and D, ‘peaks from concordant genes’ in the differentially downregulated expression category are peaks that decrease for genes that similarly decrease their expression in response to signal.

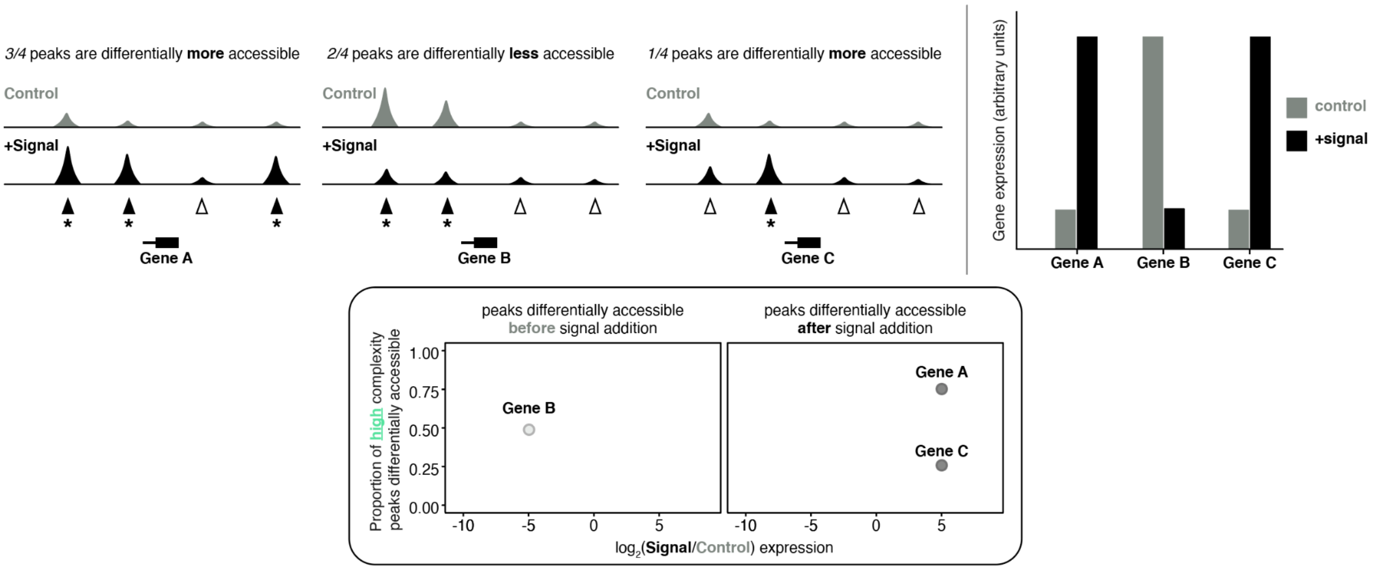

- Did the authors try to separate promoter-proximal from really distal (>20 kbp) ATAC peaks and redo the analysis to see if the relative enrichment changes?

We have not performed such an analysis, but thank the reviewer for the useful suggestion. Similar to our ‘Window’ analysis, we performed a similar analysis using promoter proximal defined peaks, which we defined as those within 1.5 kilobases up or downstream of the transcription start site. Further, we also looked at only distal peaks by taking peaks that were greater than 20 kilobase pairs from the transcriptional start site but fewer than 50 kilobase pairs from the transcription start site. We have updated the text to reflect these findings and the changes are shown in updated text in response to Reviewer 1’s comment number 4 and have included these analyses in Supplemental Figures 16A,B.

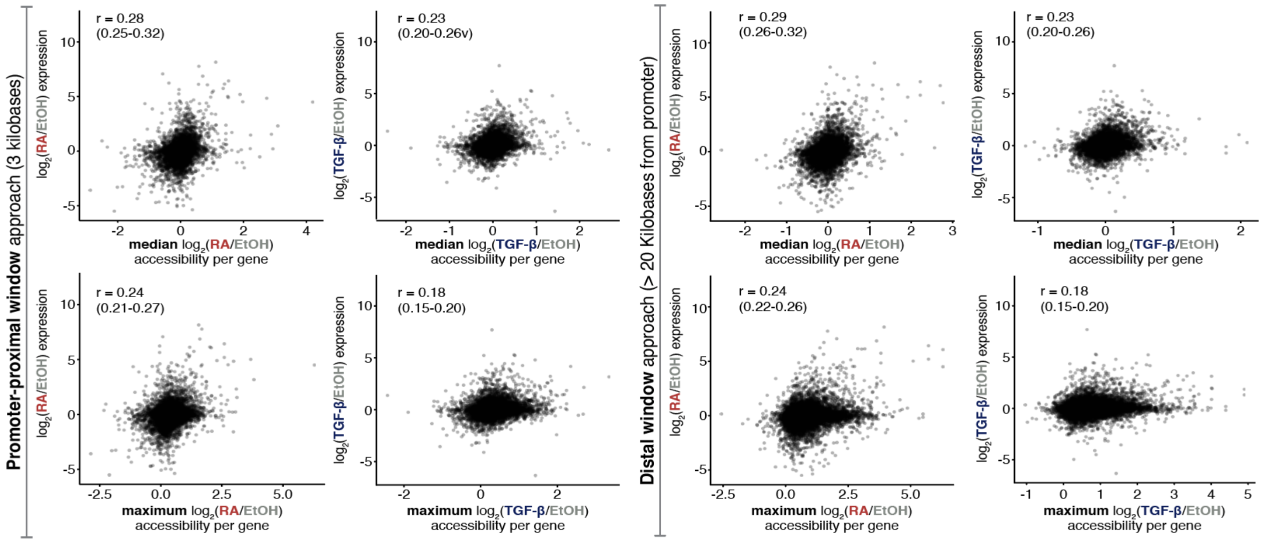

Promoter-proximal window approach (3 kilobases)
Distal window approach (> 20 Kilobases from promoter)
r = 0.28
(0.25-0.32)
r = 0.23
(0.20-0.26v)
r = 0.29
(0.26-0.32)
r = 0.23
(0.20-0.26)
r = 0.24
(0.21-0.27)
r = 0.18
(0.15-0.20)
r = 0.24
(0.22-0.26)
r = 0.18
(0.15-0.20)
log2(RA/EtOH) expression
log2(TGF-β/EtOH) expression
median log2(RA/EtOH)
accessibility per gene
median log2(TGF-β/EtOH)
accessibility per gene
maximum log2(RA/EtOH)
accessibility per gene
maximum log2(TGF-β/EtOH)
accessibility per gene

Reviewer #2:

The major goal of this manuscript is to investigate the concordance between gene expression and chromatin accessibility. To do this, the authors analyzed data from MCF-7 cells which were subjected to two different environmental stimulations (retinoic acid and TGF-beta) and paired ATAC-seq/RNA-seq from a hematopoietic different. From these data, the authors observed two classes of genes: 1) genes whose expression changes were accompanied by changes in local chromatin accessibility and, 2) genes which no change in chromatin accessibility could be detected, concluding that gene expression changes can occur irrespective of chromatin accessibility changes.

While the conclusions are not surprising -- TFs that drive chromatin accessibility and not necessarily the drivers of function -- this study represents a strong framework to resolve the qualitative and quantitative relationship between chromatin accessibility and gene expression. Overall, I have no concern with respect with major conclusions of the manuscript however, I think the presentation (figures) complicate the relatively simple conceptual findings. In particular, I found Fig 2C,D,E (left panels) and Fig 3 (some of the main conclusions of the manuscript) challenging to interpret.

> Thank you for the feedback, in order to make the latter panels of Figure 2 more clear, we have added a schematic (now Figure 2A, shown above in response to a minor comment from Reviewer 1) to contextualize how the raw data were used to create these plots with example data in order to make interpretation of the figure more clear. Furthermore, we have changed the labeling in Figure 3 panels C and D and Supplement Figure 12 panels C and D to make the comparisons we examined more clear.

Comments/concerns:

1. Introduction, paragraph 4: "it is unclear how well accessibility-based methods capture activity of all transcription factors. It is possible that some transcription factors' binding and activity does not result in corresponding changes in accessibility and vice-versa." The authors briefly address the previous work studying the binding of TFs with respect to open chromatin (John et al. Nat. Genetics 2011). It has been shown that GR/ER binds almost exclusively to sites with preexisting chromatin accessibly despite considerable changes in gene expression. In a follow-up study from Gordon Hager's group (Biddie et al. Mol. Cell 2011) they show that these sites are bookmarked by AP-1 that maintains accessibility in the absence of an environmental stimulus. It would be useful for the authors to contextualize these previous findings in the introduction. More broadly, a discussion about the environmental stimulation in cell lines vs. cellular differentiation would be helpful.

> Thank you for the citation and suggestion. We have updated the introduction with a discussion of the findings of these two papers along with a broader discussion of environmental stimulation vs. cellular differentiation as shown below:
>
> > However, it is unclear how well these accessibility based methods capture the activity of all transcription factors. It is possible that some transcription factors' binding and activity does not result in corresponding changes in accessibility and vice versa. Such a lack of correspondence could manifest itself as a lack of correlation between changes in accessibility and changes in transcription. Given the underlying assumption that a change in transcription must be mediated by the change in some transcription factor activity, then such a lack of correspondence would suggest that changes in the activity of transcription factors could change expression without changing accessibility near its binding site. Indeed, previous work has demonstrated that the glucocorticoid receptor binds almost exclusively to pre-existing accessible chromatin prior to small-molecule stimulation (John et al. 2011), and

that activator protein 1 (AP-1) establishes this binding patterns for the glucocorticoid receptor by maintaining chromatin accessibility (Biddie et al. 2011). Similarly, the lineage-defining transcription factor Foxp3, binds to preformed accessible sites established by its structural homolog, Foxo1, to establish regulatory T cell identity (Samstein et al. 2012). Of note, this process of regulatory T cell specification via Foxp3 is considered a 'late differentiation' process, as the precursor cell state, the mature naive $CD4^{+}$ T cell is considered mature. While reports from the literature generally show a strong correspondence (de la Torre-Ubieta *et al*, 2018; González *et al*, 2015; Ampuja *et al*, 2017; Starks *et al*, 2019), it is worth noting that the comparisons in such studies are often across rather different cell types. In such cases, it is possible that the changes in accessibility are not driven by regulation *per se*, but rather reflect the consequences of sequential exposure to multiple regulatory factors that characterize the differentiation process. Such accessibility changes could, in principle, signify the reinforcement of genes that are already transcriptionally active genes, or could even just appear around actively transcribed genes without any functional role. Disentangling such possibilities could be revealed with the use of single-factor perturbations that more directly affect an individual pathway; however, few such data are available.

2. Fig 1C. I find the breakdown of differentially accessibility sites and expression over-simplifies the likely reality of the data. How many ATAC-peaks are assigned to each gene? How many ATAC-seq peaks have reduced accessibly, or conversely increased accessibility. How many genes are associated with two or more ATAC-seq peaks which have divergent changes in accessibility vs. genes with concordant changes in chromatin accessibility? How do you deal with the case where a gene is associated with both up-regulated and down-regulated ATAC/DNase I peaks?

We thank the reviewer for the feedback and apologize for any lack of clarity regarding the data in figure 1C. To address this, we have added a new figure (updated Supplemental Figures 1,2) with these data. We have updated the main text to both reflect the results of these analyses and to clarify how we dealt with both up and down-regulated ATAC/DNase I peaks. We have also updated the methods to further clarify dealing with cases where a gene is associated with both up and down-regulated peaks.

Differential gene expression and differential peak accessibility analysis showed a dose-dependent response to both signals compared to ethanol control (Figure 1A and Supplemental Figure 1A, bar plots). The ethanol 'vehicle' controls comprise three different densities of cells, and the transcriptomes of control conditions globally were similar regardless of cell density (Supplemental Figure 1B). To confirm that global gene expression and chromatin accessibility patterns were similar between signals and dosages, we performed principal component analysis. For both RNA-seq and ATAC-seq data, all samples exposed to the same signal or ethanol control clustered together, indicating that their gene expression and chromatin accessibility were more similar to each other than to other conditions, supporting the quality of these data.

We next wondered whether genes that were differentially expressed were more likely to have differentially accessible peaks nearby, i.e., was there concordance between gene expression and chromatin accessibility changes at the level of individual genes? To initially characterize the extent of concordance between these data, we looked at the overlap between genes that were differentially expressed in response to high dose signal and genes with differentially accessible peaks nearby after exposure to signal (Figure 1C).

We assigned each accessible peak to the nearest transcriptional start site ("nearest approach"). Using this approach, the majority of genes had fewer than 20 peaks assigned to

them (Supplemental Figure 2A). and found that of the over 2000 genes upregulated in response to high dose retinoic acid, more than half of them had at least one differential peak (irrespective of the direction of peak change) assigned to its transcriptional start site (p-value < $2.2x10^{-16}$, Fisher's exact test). Similarly, a third of the genes whose expression was upregulated in response to TGF-β had differential peaks assigned to them (p-value < $2.2x10^{-16}$, Fisher's exact test). For differentially expressed genes in response to high dose retinoic acid or TGF-β, approximately 75% and 81% of genes had all peaks either not differentially accessible or differentially accessible in the same direction of gene expression changes, respectively (Supplemental Figure 2B). Thus, using the 'nearest' approach, genes that are differentially expressed are more likely than random chance to have a nearby peak that is differentially accessible in response to retinoic acid or TGF-β.

Methods:

We employed two methods for assigning peaks to genes. In the 'nearest' approach, we used annotation from ChIPseeker to assign each peak to the nearest transcriptional start site. With this method, each peak is uniquely mapped to a single gene. In the 'window' approach we used a window of 50 kilobases on either side of the transcriptional start site (100 kilobases in total) to assign peaks to a gene, which could result in a peak being assigned to multiple genes.

For the initial overlap analysis for Figure 1, peaks were assigned to genes using the 'nearest method.' Overlap was considered if any of the differentially expressed genes had a differentially accessible peak, regardless of whether the expression and accessibility changes were in the same direction. Further analyses integrating gene expression and chromatin accessibility data took magnitude and direction of change into account.

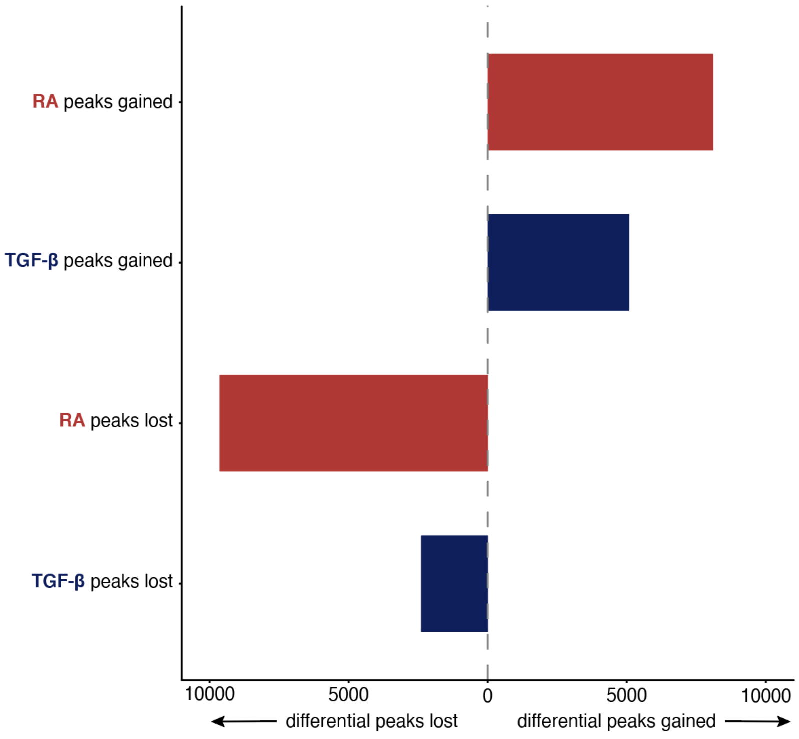

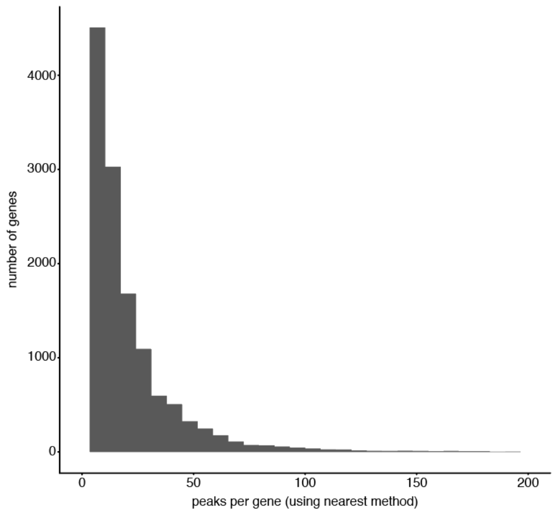

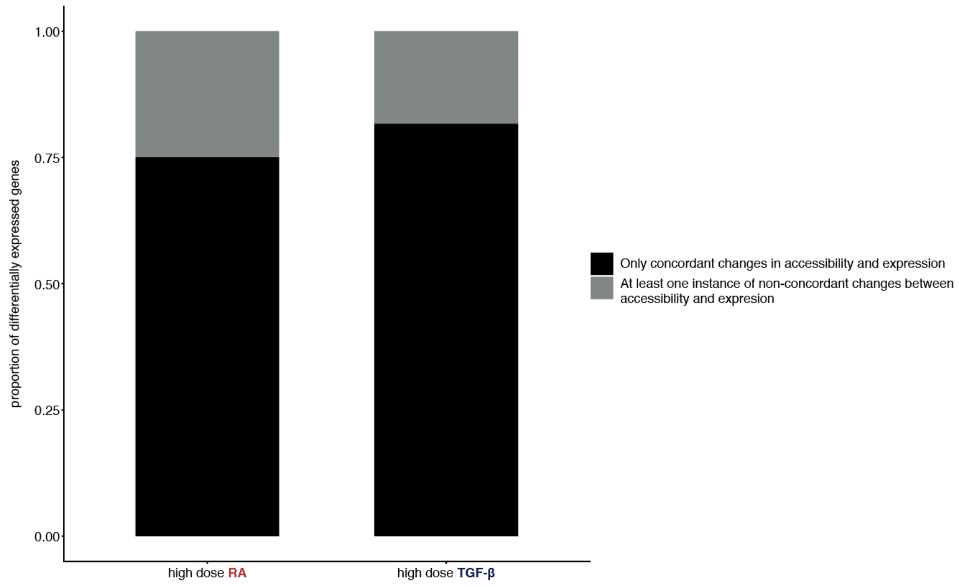

3. Fig 1B; right panel (motifs). The authors should note the motifs shown represent only 4 distinct recognition sequences (RARA, HOX, SMAD and AP1 (TGAgTCA)).

We thank the reviewer for making this distinction, we have made note of this in the methods.

> We performed motif analysis on our set of differential peaks using chromVAR v1.8.0 (Schep *et al*, 2017), its associated cisBP database of transcription factor motifs, and the motifmatchR package from bioconductor. To decrease the variance of the transcription factor motif deviations scores, we pooled together the different dosages of retinoic acid or TGF-β. The chromVAR code was modified to extract an internal metric that equals the fractional change in fragment counts at motif-containing peaks for a given motif. For the calculation of motif enrichment scores, the motifs we used were derived from four distinct groups of motif recognition sequences: RARA, HOX, SMAD, and AP1.

4. Fig 1C. I find the breakdown of differentially accessibility sites and expression over-simplifies the likely reality of the data. How many ATAC-peaks are assigned to each gene? How many ATAC-seq peaks have reduced accessibility, or conversely accessibility. How many genes are associated with two or more ATAC-seq peaks which have divergent changes in accessibility vs. genes with concordant changes in chromatin accessibility?

Please see above for point 2

5. "We observed that approximately 55% of peaks from hematopoietic differentiation data overlapped with peaks from the MCF-7 signal response data". Does the 55% refer to all peaks, irrespective if they are classified as differentially accessible? In any case, I am not convinced that qualitative comparison of the ATAC/DNaseI windowed density at three loci demonstrates that systematic differences are not present. In fact, the authors noted in the previous paragraph (and in Supplementary Fig. 2B) that there exist quantifiable differences in the genomic distribution of differentially accessible peaks between the DNase and ATAC data (possible promoter bias for ATAC). How many total reads map to promoter proximal peaks vs. distal sites in each dataset?

- Clarify the overlapping peaks point made with respect to comparing hematopoietic DHS to ATAC data. Also, compare number of reads mapping to promoter proximal vs distal sites in each dataset

  We thank the reviewer for this comment and apologize for any lack of clarity regarding this point. The 55% value refers to all peaks (both differentially accessible and not) and was calculated using bedtools intersect to demonstrate the degree of overlap between all peaks from one study and all peaks from the hematopoietic differentiation data set. We have updated the text (below), to more clearly explain this. Moreover, while there indeed is a difference between the relative proportion of accessible features between DNase-seq and ATAC-seq as demonstrated in Supplementary Figure 6B (formerly Supplementary Figure 2B), we attempt to somewhat correct for this in Figure 4C where we look at the relationship between the magnitude of change in accessibility and the magnitude of change in gene expression by subsetting accessible peaks based on their the location in relation to gene bodies. This analysis showed that for both data sets that the quantitative relationship was strongest between peaks at the promoter, however the overall qualitative result was similar across regions. Further, in response to a comment by Reviewer 3, we further looked at paired ATAC-seq and RNA-seq data set from a directed differentiation of HL-60 promyelocytes to monocyte-derived macrophages from Ramirez et al., 2017 which demonstrates stronger concordance than our data from MCF-7 cells responding to retinoic acid or TGF-β, indicating that the strong concordance from the hematopoietic differentiation data set cannot be explained by a difference in the technique used to measure accessibility.

> Moreover, to look at similarities in accessibility genome-wide, we calculated the intersection of the consensus peak sets from hematopoietic differentiation and MCF-7 signal response data sets, which included both peaks that were differentially accessible and those that were not. We observed that approximately 55% of peaks from hematopoietic differentiation data (DNase-seq) overlapped with peaks from the MCF-7 signal response data set (ATAC-seq). These results show that the datasets do not have systematic qualitative differences in either expression or accessibility, enabling us to compare the degree of concordance across these two systems.

6. Supplementary Fig 5B. It is not possible to distinguish the peak widths of low complexity vs. high complexity (teal vs. green). I suggest a different way to compare these two classes (such as a boxplot).

  Thank you for the suggestion, we have faceted the data in Supplemental Figure 7D (what was formerly Supplementary Figure 5B) to delineate more clearly the data for low and high complexity peaks.

D

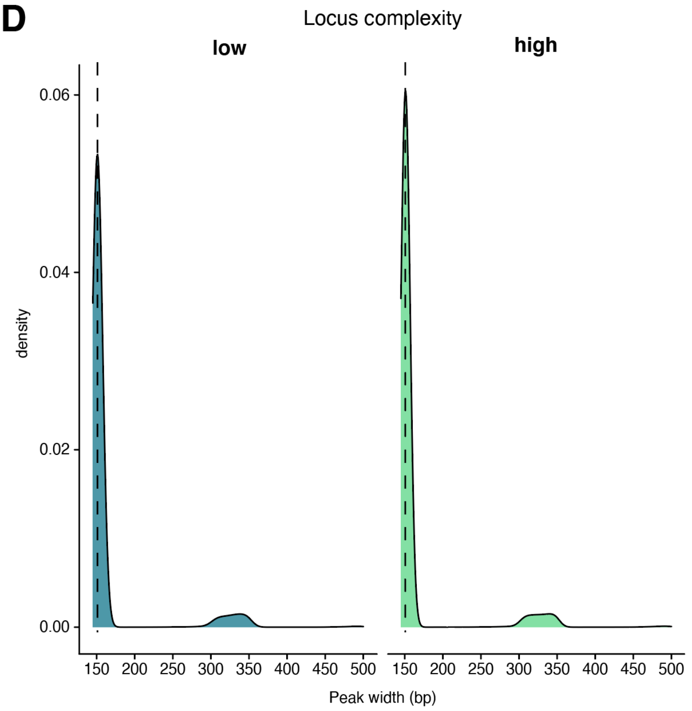

7. Pg 6. "By comparison, the distribution was skewed toward more (lower complexity) genes having a lower proportion of peaks being differentially accessible in the concordant direction in response to signals in MCF-7 cells, especially in the case of TGF-β". Is this expected, given that the total number of differentially accessible peaks in the hematopoietic differentiation data is ~2X larger than the MCF-7 differential peaks?

> We thank the reviewer for raising this point about our claim regarding associations and the potential for systematic differences in our dataset. While we are referring to proportionality, which should in principle normalize such differences, and did not see other systematic differences in the dataset, the reviewers point is very well taken. We have added the following caveat:
>
> > By comparison, the distribution was skewed toward more (lower complexity) genes having a lower proportion of peaks being differentially accessible in the concordant direction in response to signals in MCF-7 cells, especially in the case of TGF-β. However, it is also possible that these differences may owe to systematic differences between the datasets, given that the hematopoietic differentiation data have twice as many differentially accessible peaks as the MCF-7 data.

8. Fig 3A/B. From my understanding of the figure legend the y-axes refer to the proportion of ATAC/DNase I peaks that have concordant changes (i.e., gene expression increases, ATAC/DNAse I peak increases). The value that distinguishes low proportion of concordant accessibility vs. changes to its peaks appears to be 10% (dotted line in figure panel). What is the rationale behind this threshold value?

> We thank the reviewer for noticing this discrepancy, and we apologize for any confusion we have caused. The previous version indeed had an incorrect threshold. We have updated the figure to reflect our method of thresholding at a proportion of peak change at zero. We have also updated the associated analyses from Figure 3C,D and Supplemental Figure 12C,D to reflect reallocating peaks according to this threshold.

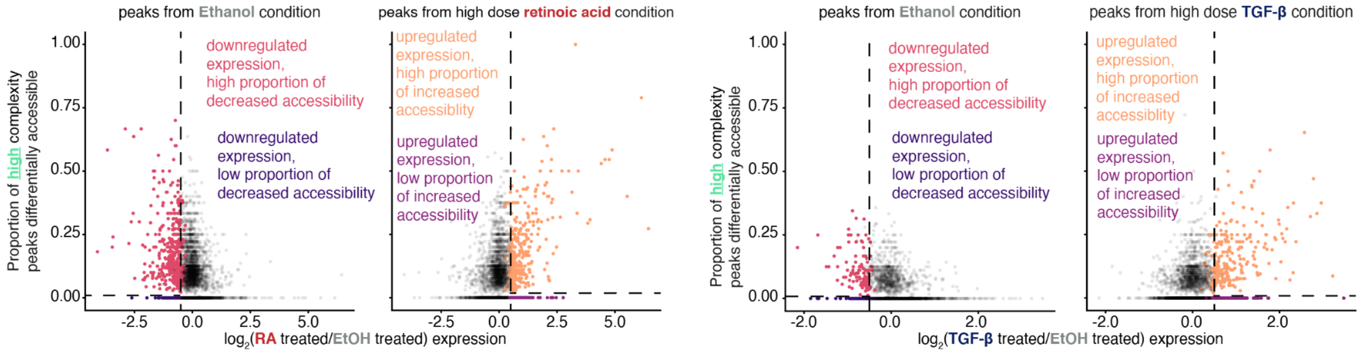

9. Supplementary Fig 4. panels are not labelled.

Thank you for the suggestion, we have added labels for the panels and formatted all figures per journal guidelines.

Reviewer #3:

This manuscript by Kiani et al utilizes previously published paired chromatin accessibility and RNA expression datasets to address the question of the association between changes in RNA expression and chromatin accessibility. Using these datasets they conclude that many changes in RNA expression are not associated with changes in chromatin accessibility, a finding that is more evident with their single-factor perturbation data as opposed to the cellular differentiation datasets. In addition, they categorize this finding based on genes with more neighboring regulatory elements. Overall, the conclusions drawn by the authors are not entirely surprising based on prior studies that have looked into this finding. However, their conclusion regarding the differences between single-factor perturbation data as opposed to the cellular differentiation datasets is of interest. Below are some comments.

We thank the reviewer for their careful evaluation of our manuscript, and are encouraged that they found aspects of our work to be of interest.

The abstract makes a statement regarding a causal relationship between changes in accessibility and changes in expression. Specifically, it says that "some changes to accessibility changes may occur after changes to expression". As the datasets that they are evaluating are only for two time points (i.e., before and after treatment), it is quite challenging to ascribe causality. Also, I am unsure where in the manuscript that this conclusion is being derived from.

We thank the reviewer for pointing this issue out, and we agree. We should note that we were not referring to comparisons within a dataset (which we do not have), but rather between the hematopoiesis and MCF-7 datasets. That said, we agree that this claim is a bit tenuous, and so we have now removed it from the abstract entirely.

> Together, these results show many gene expression changes can happen independent of changes in accessibility of local chromatin in the context of a single-factor perturbation ~~and suggest that some changes to accessibility changes may occur after changes to expression, rather than before~~.

The authors should spend more time discussing the John et al., paper (PMID 21258342) and Samstein et al paper (PMID 23021222) in the introduction. These papers serve as a foundation in terms of how single-factor perturbations and cellular differentiation impact chromatin accessibility profiles. A lot of the findings regarding chromatin accessibility that the authors present in this manuscript are documented in these prior studies.

Thank you for suggesting the cited study, we have integrated a discussion of these two papers as well as one paper suggested by reviewer 2 into the introduction. Please refer to our response to comment 1 from reviewer 2 to see the changes we have made to the introduction adding a discussion of these papers.

The structure of this manuscript could be significantly improved. It felt as though it was jumping around a lot, and was quite verbose for what in the end is a rather simple conclusion.

We apologize for any confusion or lack of clarity caused by the structure of our manuscript. We agree that our manuscript is perhaps a bit verbose and technical in some places, but we felt that given our goal of supporting our claim strongly, we wanted to transparently discuss the technical nuances of our study. We appreciate the reviewer's point, however, and thus have expanded the last paragraph of the introduction to better serve as an overview of the analyses we perform to

better clarify the organization of the paper. We believe this helps orient the reader and thank the reviewer for pointing this issue out.

> Here, we used tandem bulk RNA-seq and ATAC-seq data from MCF-7 breast carcinoma cells exposed to multiple doses of retinoic acid or TGF-β to determine the degree of concordance between changes in chromatin accessibility and changes in gene expression. We demonstrate that while some differentially expressed genes have a high concordance between gene expression and chromatin accessibility changes, many other genes are differentially expressed without changes in their local chromatin accessibility. ~~Furthermore, we evaluated concordance in another published data set of hematopoietic differentiation to validate our approach based on well-defined and specific perturbations.~~ We show that these results hold across multiple parameters and definitions of accessibility change, and that it does not depend on the type of transcription factor per se. We evaluated another published data set of hematopoietic differentiation, which has much deeper and multi-factorial differences, that showed much stronger concordance. We finally compare differences in pre-existing accessibility between concordant and non-concordant genes prior to single-factor perturbation. Our results provide a systematic evaluation of the concordance between changes in gene expression and local chromatin accessibility.

Figure 1E. Can you please show the mapped RNA reads on this browser figure. It is unclear where these RNA reads are coming from, as the transcriptional start site in the annotated gene does not appear to be accessible in the provided plot.

Thank you for the suggestion, we have added a track with the mapped RNA reads. To make this distinction clear and avoid overcrowding of the figure, we have made a supplemental figure (Supplemental Figure 4) to show ATAC-seq fragment counts per million as well as RNA fragment counts between ethanol and high dose retinoic acid conditions.

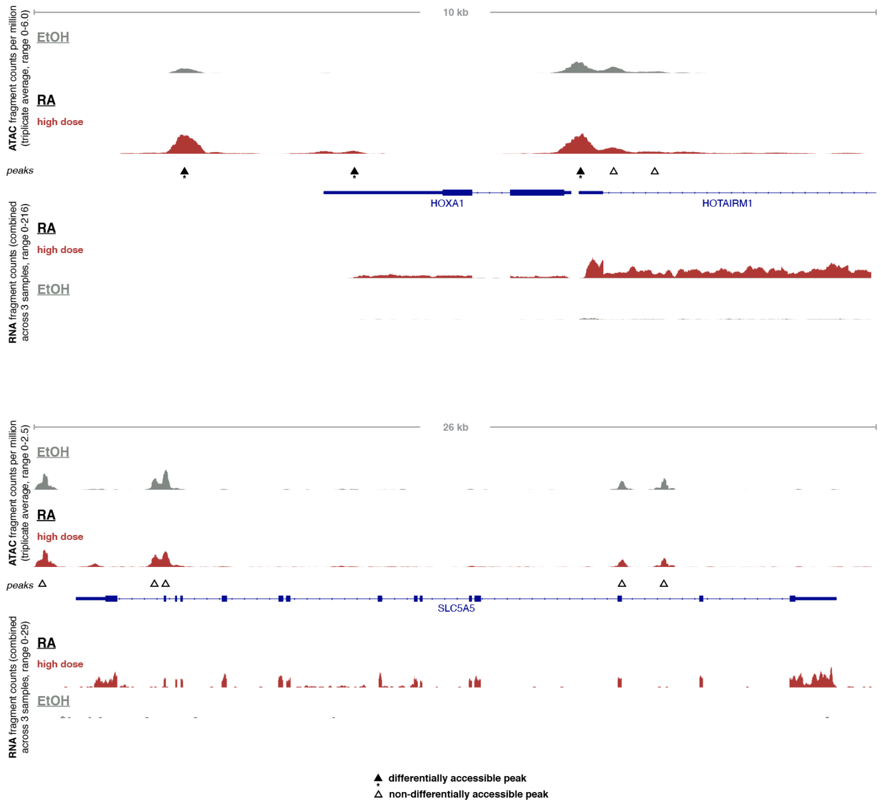

Figure 1C. Please correct the Venn diagrams to make the individual circles appropriately scaled.

> Thank you for the suggestion, we have scaled the circles from the two Venn diagrams in Figure 1C to clarify our point.

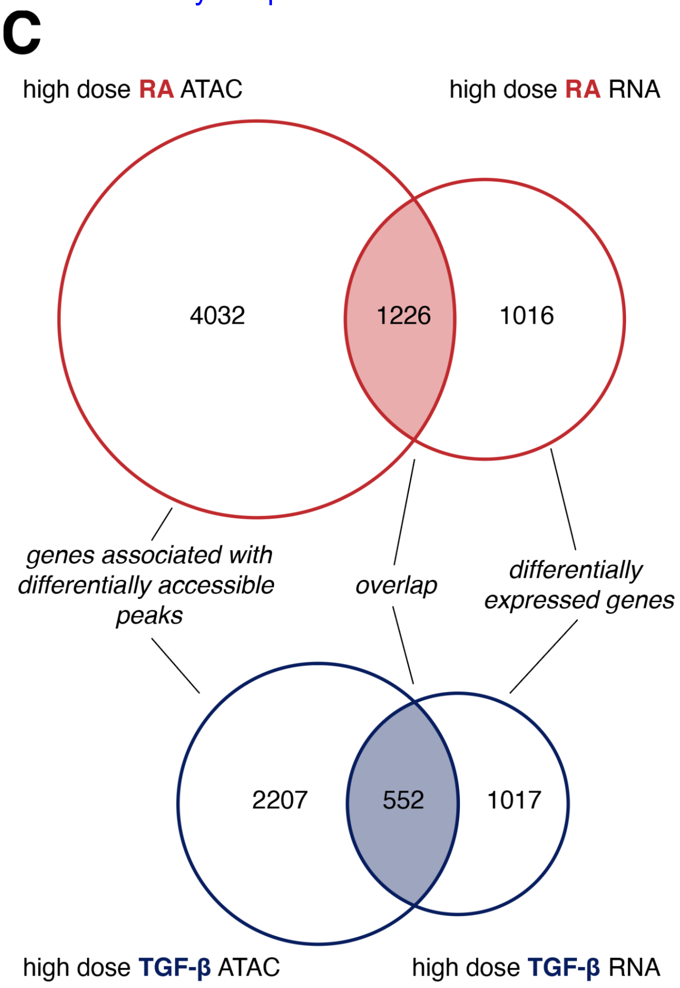

The data presented in Sup Figure 8 does not appear to support the conclusion "We quantified the distribution of peak complexity across these groups and observed that they were similar across all four gene subgroups (Supplemental Figures 8A,B)". Specifically, it appears that the "upregulated expression, high proportion of increased accessibility" has significantly higher gene complexity than the "upregulated expression, low proportion of increased accessibility". Please provide p-values for the comparisons on this plot and correct this statement accordingly.

> Thank you for pointing this out, we have updated supplemental Figure 12 A and B (Formerly Supplemental Figure 8) and have updated the text to reflect these changes.

>> We wondered what the differences were between genes that were differentially expressed and had large accessibility changes versus those that were differentially expressed and had low accessibility changes. First, for high dose retinoic acid and TGF-β, we split genes into four groups based on whether they were differentially expressed and the proportion of peaks assigned to them using the 'nearest' method that were differentially accessible in the appropriate direction. These four groups were (1) genes with differentially upregulated expression and concordant accessibility changes (2) genes with differentially upregulated expression non-concordant accessibility changes (3) genes with differentially downregulated expression and a concordant accessibility changes, and (4) genes with with differentially downregulated expression and non-concordant accessibility changes (Figures 3A,B). We

quantified the distribution of peak complexity across these groups and observed that genes with any concordant peaks for both conditions in both directions tended to have a higher degree of locus complexity. ~~they were similar across all four gene subgroups~~ (Supplemental Figures 12A,B).

The authors mention differences in the distribution of promoter versus distal peaks in the ATAC-seq and DNAseI-seq datasets that they are using. ATAC-seq has a known bias towards promoters, so this is not surprising. The authors try to correct for this in Figure 4C, but they should further comment on whether this difference could explain the difference they are seeing between the single-factor perturbation data as opposed to the cellular differentiation datasets? Are there differentiation datasets they can use that are also ATAC-seq?

We thank the reviewer for the suggestion. In order to address the point of whether the difference in genome-wide accessibility measurement technique (DNase- versus ATAC-seq) and their relative bias for or against reads at the promoter, respectively, we used a data set from Ramirez et al., 2017, PMID: 28365152, which had paired RNA- and ATAC-seq data of HL-60 promyelocytes before and after a two step differentiation to monocyte-derived macrophages. Using a similar to analysis as Figure 2 where we plot change in gene expression against the proportion of peaks with concordant changes, we show that there is more concordance than with the single-factor perturbation data, indicating that the difference in findings we observed between single-factor and differentiation data are likely not due to technical differences in the method of measuring chromatin accessibility. These findings further highlight the need for further systematic analyses in a wide variety of experimental contexts.

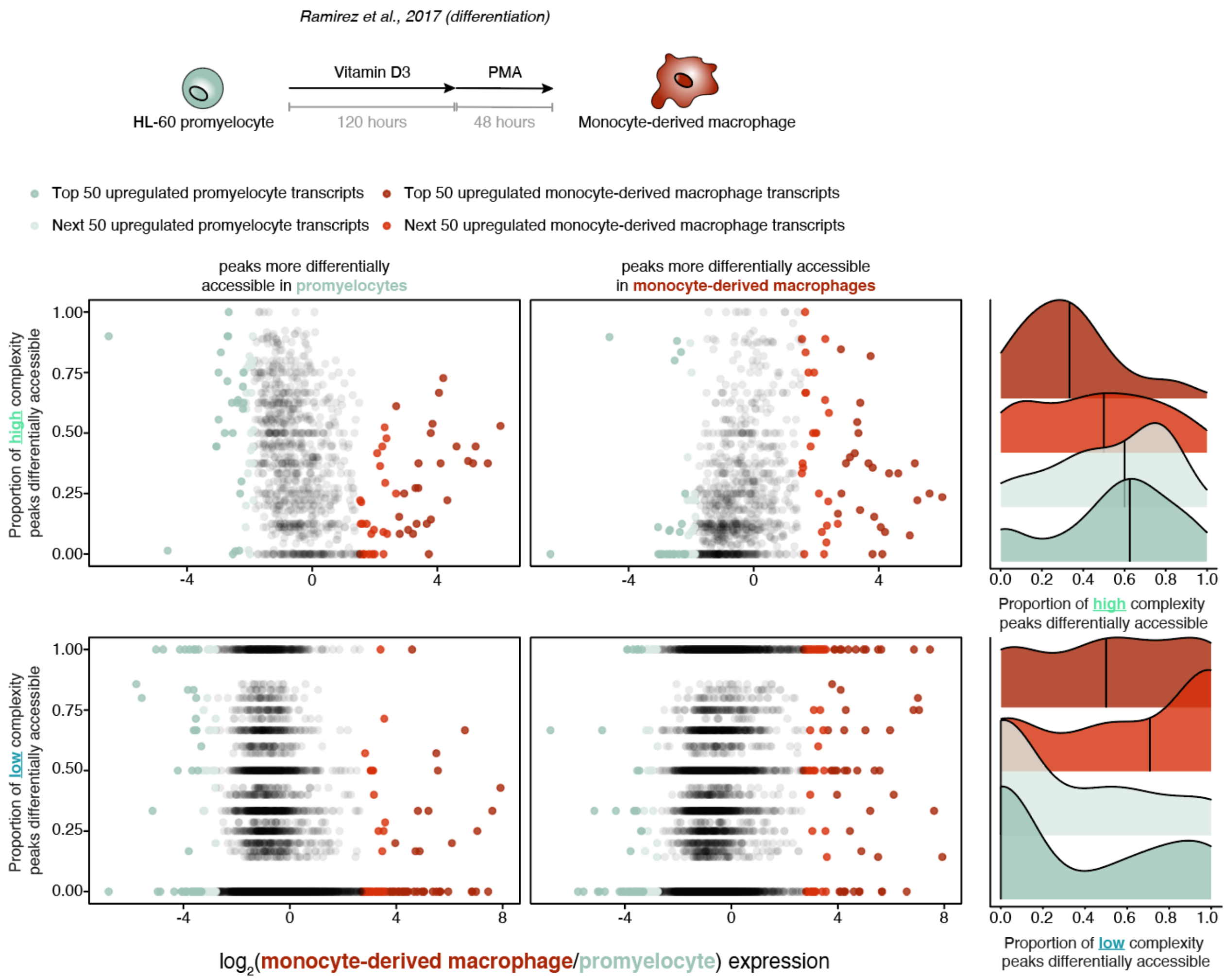
Ramirez et al., 2017 (differentiation)
Vitamin D3
PMA
HL-60 promyelocyte
120 hours
48 hours
Monocyte-derived macrophage
Top 50 upregulated promyelocyte transcripts
Top 50 upregulated monocyte-derived macrophage transcripts
Next 50 upregulated promyelocyte transcripts
Next 50 upregulated monocyte-derived macrophage transcripts
peaks more differentially accessible in promyelocytes
peaks more differentially accessible in monocyte-derived macrophages
Proportion of high complexity peaks differentially accessible
Proportion of low complexity peaks differentially accessible
log2(monocyte-derived macrophage/promyelocyte) expression

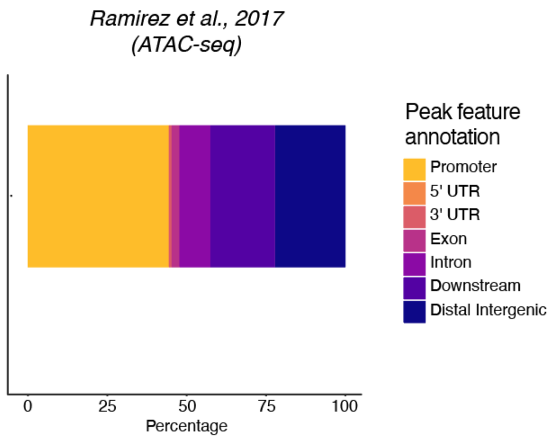
Ramirez et al., 2017 (ATAC-seq)
Peak feature annotation
Promoter
5' UTR
3' UTR
Exon
Intron
Downstream
Distal Intergenic
Percentage

1st Revision - Editorial Decision 22nd Jul 2022

Manuscript Number: MSB-2022-10979R
Title: Chromatin accessibility changes are not concordant with transcriptional changes for single signals

Thank you for sending us your revised manuscript. We have now heard back from the reviewer who was asked to evaluate your revised study. As you will see below, the reviewer is satisfied with the performed revisions and is supportive of publication.

Before we formally accept the study for publication we would ask you to address some remaining editorial issues listed below.
Reviewer #1:

I find that the authors addressed all outstanding remarks by me (and by the other reviewers) very thoroughly. The results of their analysis are now well presented and approached from many different angles; the manuscript has really improved in clarity and I recommend publication in its current form in MSB. The work will be of interest for many of us that use chromatin accessibility data as an indicator of changing gene activity.

A. Papantonis

Dear Dr. Polychronidou,

We are delighted to hear the positive response to our resubmission of “Chromatin accessibility changes are not concordant with transcriptional changes for single signals.” We have addressed the following points in response to the editorial team and reviewers:

All changes in the manuscript .docx file were made in track changes mode to address the above comments.

Please feel free to contact us if there is any additional information we can provide, and thank you again for your guidance in this process.

Sincerely,

Arjun Raj

Manuscript number: MSB-2022-10979RR
Title: Chromatin accessibility changes are not concordant with transcriptional changes for single signals

Thank you again for sending us your revised manuscript. We are now satisfied with the modifications made and I am pleased to inform you that your paper has been accepted for publication.

EMBO Press Author Checklist

Corresponding Author Name: Arjun Raj
Journal Submitted to: Molecular Systems Biology
Manuscript Number: MSB-2022-10979

USEFUL LINKS FOR COMPLETING THIS FORM
The EMBO Journal - Author Guidelines
EMBO Reports - Author Guidelines
Molecular Systems Biology - Author Guidelines
EMBO Molecular Medicine - Author Guidelines

Reporting Checklist for Life Science Articles (updated January
This checklist is adapted from Materials Design Analysis Reporting (MDAR) Checklist for Authors. MDAR establishes a minimum set of requirements in transparent reporting in the life sciences (see Statement of Task: 10.31222/osf.io/9sm4x). Please follow the journal's guidelines in preparing your
Please note that a copy of this checklist will be published alongside your article.

Abridged guidelines for figures
1. Data
The data shown in figures should satisfy the following conditions:
- the data were obtained and processed according to the field's best practice and are presented to reflect the results of the experiments in an accurate and unbiased manner.
- ideally, figure panels should include only measurements that are directly comparable to each other and obtained with the same assay.
- plots include clearly labeled error bars for independent experiments and sample sizes. Unless justified, error bars should not be shown for technical
- if n<5, the individual data points from each experiment should be plotted. Any statistical test employed should be justified.
- Source Data should be included to report the data underlying figures according to the guidelines set out in the authorship guidelines on Data

2. Captions
Each figure caption should contain the following information, for each panel where they are relevant:
- a specification of the experimental system investigated (eg cell line, species name).
- the assay(s) and method(s) used to carry out the reported observations and measurements.
- an explicit mention of the biological and chemical entity(ies) that are being measured.
- an explicit mention of the biological and chemical entity(ies) that are altered/varied/perturbed in a controlled manner.
- the exact sample size (n) for each experimental group/condition, given as a number, not a range;
- a description of the sample collection allowing the reader to understand whether the samples represent technical or biological replicates (including how many animals, litters, cultures, etc.).
- a statement of how many times the experiment shown was independently replicated in the laboratory.
- definitions of statistical methods and measures:
  - common tests, such as t-test (please specify whether paired vs. unpaired), simple χ2 tests, Wilcoxon and Mann-Whitney tests, can be unambiguously identified by name only, but more complex techniques should be described in the methods section;
  - are tests one-sided or two-sided?
  - are there adjustments for multiple comparisons?
  - exact statistical test results, e.g., P values = x but not P values < x;
  - definition of 'center values' as median or average;
  - definition of error bars as s.d. or s.e.m.

Please complete ALL of the questions below.
Select "Not Applicable" only when the requested information is not relevant for your study.

Materials

Newly Created Materials	Information included in the manuscript?	In which section is the information available? (Reagents and Tools Table, Materials and Methods, Figures, Data Availability Section)
New materials and reagents need to be available; do any restrictions apply?	Not Applicable	

Antibodies	Information included in the manuscript?	In which section is the information available? (Reagents and Tools Table, Materials and Methods, Figures, Data Availability Section)
For **antibodies** provide the following information:<br>- Commercial antibodies: RRID (if possible) or supplier name, catalogue number and or/clone number<br>- Non-commercial: RRID or citation	Not Applicable	

DNA and RNA sequences	Information included in the manuscript?	In which section is the information available? (Reagents and Tools Table, Materials and Methods, Figures, Data Availability Section)
**Short novel DNA or RNA including primers, probes**: provide the sequences.	Not Applicable	

Cell materials	Information included in the manuscript?	In which section is the information available? (Reagents and Tools Table, Materials and Methods, Figures, Data Availability Section)
**Cell lines:** Provide species information, strain. Provide accession number in repository **OR** supplier name, catalog number, clone number, and/**OR** RRID.	Not Applicable	
**Primary cultures:** Provide species, strain, sex of origin, genetic modification status.	Not Applicable	
Report if the cell lines were recently **authenticated** (e.g., by STR profiling) and tested for mycoplasma contamination.	Not Applicable	

Experimental animals	Information included in the manuscript?	In which section is the information available? (Reagents and Tools Table, Materials and Methods, Figures, Data Availability Section)
**Laboratory animals or Model organisms:** Provide species, strain, sex, age, genetic modification status. Provide accession number in repository **OR** supplier name, catalog number, clone number, **OR** RRID.	Not Applicable	
**Animal observed in or captured from the field:** Provide species, sex, and age where possible**.**	Not Applicable	
Please detail **housing and husbandry conditions**.	Not Applicable	

Plants and microbes	Information included in the manuscript?	In which section is the information available? (Reagents and Tools Table, Materials and Methods, Figures, Data Availability Section)
**Plants:** provide species and strain, ecotype and cultivar where relevant, unique accession number if available, and source (including location for collected wild specimens).	Not Applicable	
**Microbes:** provide species and strain, unique accession number if available, and source.	Not Applicable	

Human research participants	Information included in the manuscript?	In which section is the information available? (Reagents and Tools Table, Materials and Methods, Figures, Data Availability Section)
If collected and within the bounds of privacy constraints report on age, sex and gender or ethnicity for all study participants.	Not Applicable	

Core facilities	Information included in the manuscript?	In which section is the information available? (Reagents and Tools Table, Materials and Methods, Figures, Data Availability Section)
If your work benefited from core facilities, was their service mentioned in the acknowledgments section?	Not Applicable	

Design

Study protocol	Information included in the manuscript?	In which section is the information available? (Reagents and Tools Table, Materials and Methods, Figures, Data Availability Section)
If study protocol has been **pre-registered, provide DOI in the manuscript**. For clinical trials, provide the trial registration number **OR** cite DOI.	Not Applicable	
Report the **clinical trial registration number** (at ClinicalTrials.gov or equivalent), where applicable.	Not Applicable	

Laboratory protocol	Information included in the manuscript?	In which section is the information available? (Reagents and Tools Table, Materials and Methods, Figures, Data Availability Section)
Provide DOI OR other citation details if **external detailed step-by-step protocols** are available.	Not Applicable	

Experimental study design and statistics	Information included in the manuscript?	In which section is the information available? (Reagents and Tools Table, Materials and Methods, Figures, Data Availability Section)
Include a statement about **sample size** estimate even if no statistical methods were used.	Not Applicable	
Were any steps taken to minimize the effects of subjective bias when allocating animals/samples to treatment (e.g. **randomization procedure**)? If yes, have they been described?	Not Applicable	
Include a statement about **blinding** even if no blinding was done.	Not Applicable	
Describe **inclusion/exclusion criteria** if samples or animals were excluded from the analysis. Were the criteria pre-established?<br><br>If sample or data points were omitted from analysis, report if this was due to attrition or intentional exclusion and provide justification.	Not Applicable	
For every figure, are **statistical tests** justified as appropriate? Do the data meet the assumptions of the tests (e.g., normal distribution)? Describe any methods used to assess it. Is there an estimate of variation within each group of data? Is the variance similar between the groups that are being statistically compared?	Yes	Figures, Materials and Methods

Sample definition and in-laboratory replication	Information included in the manuscript?	In which section is the information available? (Reagents and Tools Table, Materials and Methods, Figures, Data Availability Section)
In the figure legends: state number of times the experiment was **replicated** in laboratory.	Yes	Figures
In the figure legends: define whether data describe **technical or biological replicates**.	Yes	Figures

Ethics

Ethics	Information included in the manuscript?	In which section is the information available? (Reagents and Tools Table, Materials and Methods, Figures, Data Availability Section)
Studies involving **human participants**: State details of **authority granting ethics approval** (IRB or equivalent committee(s), provide reference number for approval.	Not Applicable	
Studies involving **human participants**: Include a statement confirming that **informed consent** was obtained from all subjects and that the experiments conformed to the principles set out in the WMA Declaration of Helsinki and the Department of Health and Human Services Belmont Report.	Not Applicable	
Studies involving **human participants:** For publication of **patient photos**, include a statement confirming that consent to publish was obtained.	Not Applicable	
Studies involving experimental **animals**: State details of **authority granting ethics approva**l (IRB or equivalent committee(s), provide reference number for approval. Include a statement of compliance with ethical regulations.	Not Applicable	
Studies involving **specimen and field samples:** State if relevant **permits** obtained, provide details of authority approving study; if none were required, explain why.	Not Applicable	

Dual Use Research of Concern (DURC)	Information included in the manuscript?	In which section is the information available? (Reagents and Tools Table, Materials and Methods, Figures, Data Availability Section)
Could your study fall under dual use research restrictions? Please check biosecurity documents and list of **select agents and toxins** (CDC): https://www.selectagents.gov/sat/list.htm	Not Applicable	
If you used a select agent, is the security level of the lab appropriate and reported in the manuscript?	Not Applicable	
If a study is subject to dual use research of concern regulations, is the name of the **authority granting approval and reference number** for the regulatory approval provided in the manuscript?	Not Applicable	

Reporting

The MDAR framework recommends adoption of discipline-specific guidelines, established and endorsed through community initiatives. Journals have their own policy about requiring specific guidelines and recommendations to complement MDAR.

Adherence to community standards	Information included in the manuscript?	In which section is the information available? (Reagents and Tools Table, Materials and Methods, Figures, Data Availability Section)
State if relevant guidelines or checklists (e.g., **ICMJE, MIBBI, ARRIVE, PRISMA**) have been followed or provided.	Not Applicable	
For **tumor marker prognostic studies,** we recommend that you follow the **REMARK** reporting guidelines (see link list at top right). See author guidelines, under 'Reporting Guidelines'. Please confirm you have followed these guidelines.	Not Applicable	
For **phase II and III randomized controlled trials**, please refer to the **CONSORT** flow diagram (see link list at top right) and submit the CONSORT checklist (see link list at top right) with your submission. See author guidelines, under 'Reporting Guidelines'. Please confirm you have submitted this list.	Not Applicable	

Data Availability

Data availability	Information included in the manuscript?	In which section is the information available? (Reagents and Tools Table, Materials and Methods, Figures, Data Availability Section)
Have **primary datasets** been deposited according to the journal's guidelines (see 'Data Deposition' section) and the respective accession numbers provided in the Data Availability Section?	Not Applicable	
Were **human clinical and genomic datasets** deposited in a public access-controlled repository in accordance to ethical obligations to the patients and to the applicable consent agreement?	Not Applicable	
Are **computational models** that are central and integral to a study available without restrictions in a machine-readable form? Were the relevant accession numbers or links provided?	Yes	Data availability
If publicly available data were reused, provide the respective **data citations in the reference list**.	Yes	References
